# Reviewing Additive Manufacturing Techniques: Material Trends and Weight Optimization Possibilities Through Innovative Printing Patterns

**DOI:** 10.3390/ma18061377

**Published:** 2025-03-20

**Authors:** Arturo Ramos, Virginia G. Angel, Miriam Siqueiros, Thaily Sahagun, Luis Gonzalez, Rogelio Ballesteros

**Affiliations:** 1 Facultad de Ingeniería, Universidad Autónoma de Baja California, Mexicali 21280, BC, Mexico; arturo.ramos1@uabc.edu.mx (A.R.); miriam.siqueiros@uabc.edu.mx (M.S.); thaily.sahagun@uabc.edu.mx (T.S.); luis.gonzalez18@uabc.edu.mx (L.G.); 2 Honeywell Aerospace, ETS (Engineering Test Services) Materials Laboratory, Col. Parque el Vigía No. 2, Mexicali 21395, BC, Mexico; rogelio.ballesteros@honeywell.com

**Keywords:** additive manufacturing, technology trends, bioinspired structures, lightweight structures, printing patterns

## Abstract

Additive manufacturing is transforming modern industries by enabling the production of lightweight, complex structures while minimizing material waste and energy consumption. This review explores its evolution, covering historical developments, key technologies, and emerging trends. It highlights advancements in material innovations, including metals, polymers, composites, and ceramics, tailored to enhance mechanical properties and expand functional applications. Special emphasis is given to bioinspired designs and their contribution to enhancing structural efficiency. Additionally, the potential of these techniques for sustainable manufacturing and industrial scalability is discussed. The findings contribute to a broader understanding of Additive Manufacturing’s impact on design optimization and material performance, offering insights into future research and industrial applications.

## 1. Introduction

AM technologies are among the most feasible advanced manufacturing options to create complex structures for use in technology-driven industries, such as the healthcare, such as healthcare [1], automotive [2], and aerospace industries [3].

AM has gained popularity in the media and captured the imagination of the public as well as researchers in many fields. The historical development of AM can be traced back to photo sculpture in the 1860s and topographic modeling in the 1890s. These early techniques paved the way for the ‘Photo-glyph recording’ method, patented in 1951, which utilized selective exposure of transparent photo emulsion layers during the scanning of cross-sections of the object to be reproduced [4,5].

AM generates less scrap and material waste while enabling the production of lightweight, complex structures, often hollow or porous, unlike other subtractive and formative manufacturing methods, reducing both material and energy consumption during fabrication and operation.

In addition to selecting the proper AM techniques and suitable printing parameters, the microarchitecture design of structures is one of the critical aspects of their development [6]. The advent of AM technologies has provided unique opportunities for the accurate arrangement of the sizes and internal architectures of patterns to produce complex internal architectures and passages [7,8].

Advancements in materials such as metals, polymers, composites, and ceramics have significantly broadened the scope of AM applications. Each material category offers distinct advantages: metals for structural strength, polymers for flexibility, composites for tailored properties, and ceramics for high-temperature stability [9,10]. These innovations improve the mechanical properties and functionality of AM products, enabling their use in diverse applications, including aerospace components, bioinspired designs, and medical devices.

The integration of advanced AM technologies, including binder jetting, directed energy deposition, and powder bed fusion, has further revolutionized manufacturing. These methods offer exceptional accuracy, speed, and cost-efficiency tailored to specific industrial needs [11,12]. Additionally, AM integrates bioinspired designs, mimicking natural structures to create materials with enhanced strength, adaptability, and efficiency [13,14]. This synergy of innovation is coupled with a focus on sustainability, emphasizing reduced energy consumption, waste management, and lifecycle analysis. Together, these advancements establish AM as a cornerstone of modern industry, driving innovation and enabling eco-friendly manufacturing practices [15,16].

AM-produced components demonstrate mechanical properties that are comparable to or even superior to those of traditionally manufactured parts [17] despite inherent defects such as porosity and interlayer adhesion issues. Key factors influencing strength and fatigue resistance include temperature gradients, scanning strategies, and residual stress formation. Additionally, microstructural analysis reveals significant variations in grain orientation and phase distribution, which directly impact hardness and tensile performance. Other studies have examined the reuse of metal powders in powder bed fusion (PBF) additive manufacturing, analyzing how recycling affects chemical composition, particle morphology, and microstructure [18]. Repeated thermal exposure has been found to alter powder flowability, bed density, and the properties of final parts, directly impacting manufacturability.

This ongoing development underscores the importance of further research into material behaviors and the enhancement of manufacturing methods. Such efforts are essential for developing standardized processes that can be effectively applied in key sectors that will enable AM technologies to meet the rigorous demands of industries. In this context, the focus shifts to exploring key technologies that are driving advancements in additive manufacturing and showcasing their impact on production efficiency, design flexibility, and material utilization.

Optimization techniques such as Design for Additive Manufacturing are crucial in tailoring mechanical performance by adjusting parameters like relative density, unit cell configuration, and material composition. Lattice structures have demonstrated substantial weight reductions in aerospace components [19], but challenges remain in manufacturability, material selection, and structural integrity. Research on topology optimization and hybrid lattice structures continues to advance, with emerging trends focusing on bio-inspired designs and triply periodic minimal surfaces for improved mechanical efficiency.

Laser additive manufacturing (LAM) is revolutionizing metal fabrication by achieving exceptional precision and facilitating the creation of intricate geometries. Recent advancements include multi-laser splicing for large-scale production, multi-material processing through innovative powder spreading techniques, and hybrid additive-subtractive approaches for improved surface quality [20]. Challenges such as porosity and cracking are being addressed by monitoring and process optimization. The future of LAM is currently being associated with AI integration, multi-beam laser systems, and hybrid material processing, increasing efficiency and scalability for industrial applications.

Recent studies focus on exploring the use of green laser additive manufacturing for ultra-high vacuum (UHV) applications in accelerator components [21], examining pure copper due to its high conductivity. Using a TruPrint1000 Green Edition system, the research confirms that copper membranes of 1 mm or more meet UHV standards, while thinner samples may leak. The findings highlight green laser AM as a viable method for producing thin-walled UHV components, offering design flexibility and efficiency, with further research needed to optimize processing parameters.

In the context of hydrogen technologies, AM plays a crucial role in the fabrication of Proton Exchange Membrane (PEM) fuel cells and electrolyzers. AM enables the production of PEM components with complex geometries, optimizing mass transport and electrochemical performance [22].

Advancements in multi-material printing, metal AM, and artificial intelligence (AI) integration are driving the transformation and enabling sustainable and customized manufacturing solutions. The integration of digital twins can significantly enhance AM by optimizing process efficiency, accuracy, and sustainability [23]. Implementing digital twins could improve real-time monitoring, predictive maintenance, and process simulations, allowing for better decision-making and operational flexibility, thus addressing challenges in quality control, material behavior prediction, and sustainable manufacturing. Other studies propose an adaptive framework for assessing machine learning models in data-limited Additive Manufacture applications [24]. By prioritizing influential test samples, the approach minimizes estimation bias and variance, surpassing traditional sampling methods. When applied to AM datasets, it optimizes model selection with fewer labeled samples, enhancing efficiency while preserving predictive accuracy.

Current advancements in AM, including multi-material printing, AI integration, and digital twin technology, are transforming the manufacturing landscape by enhancing efficiency, precision, and sustainability. These innovations improve existing processes while also opening new possibilities for applications across various industries. Given the rapid evolution of AM technologies, this article provides a structured overview to contextualize these advancements within the broader scope of manufacturing developments; the background section explores its historical development and technological evolution. The methods section details the main AM techniques, principles, and material considerations. Subsequently, emerging trends such as bioinspired structures, sustainability challenges, and novel applications are discussed. In conclusion, the article summarizes the main findings and suggests possible directions for future research in the field.

## 2. Background

Design and development engineers from a variety of different industries offer a fascinating range of solutions as a technological trend by increasing quality requirements in 3D printing. Most of the known industries have special quality standards and material requirements with high dimensional accuracy and reproducibility [25].

Industrial manufacturing is undergoing a significant transformation driven by the advancement of 3D printing technology. Key sectors such as the automotive, medical, and aerospace industries are driving growth by their high standards, noticing that 3D printing offers several benefits, such as faster processing times, low-cost components, and unprecedented design freedom. An example of this is Concept Laser GmbH, which has been a pioneer in laser melting technology, they have the LaserCUSING patent, a technology that generates components layer by layer using 3D CAD data. The method allows complex component production with high precision [25].

**Figure 1:**
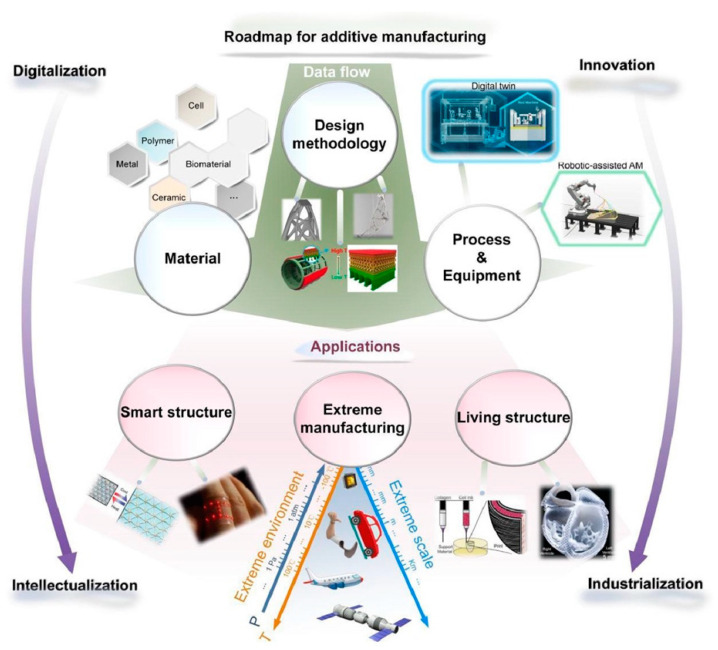
Schematic illustration of the roadmap for additive manufacturing [27].

Eyers et al. emphasize the importance of a more systematic approach in Industrial Additive Manufacturing in concept, as the current literature tends to focus on machine capabilities rather than the process itself [26]. From a different perspective, Tian et al. accentuate that the industrialization of AM marks the shift from prototyping to large-scale production by integrating automation, process control, and digital technologies. Key advancements include robotic-assisted AM, hybrid manufacturing, and real-time monitoring with advanced sensors. AI-driven optimization and digital twins enhance reliability and scalability. However, challenges remain in material consistency, multi-material printing, and post-processing. Over the next 5–10 years, efforts will focus on improving efficiency, scalability, and quality control through intelligent monitoring, big data analytics, and AI-driven design [27]. Figure 1 illustrates a roadmap for additive manufacturing (AM), highlighting the transition from digitalization to innovation and from intellectualization to industrialization.

AM is a unique technology with enormous potential. Some of the advantages highlighted by authors of the systematic approach include the availability of more extensive and documented information [26]. These enable the production of highly customized products and support on-demand manufacturing, efficiently catering to specific client needs. This significantly speeds up production cycles compared to conventional manufacturing, particularly for prototypes or small production runs. AM also facilitates the creation of complex geometries without the need for additional tooling, simplifying production processes. Furthermore, it aligns strategically with manufacturing objectives by optimizing factors such as cost, dependability, flexibility, quality, and speed. Improved resource allocation is another key advantage, as AM promotes better utilization of labor, machinery, and software tools. Additionally, material usage can be optimized, and waste can be minimized through careful planning and execution, contributing to more sustainable manufacturing practices.

Despite its potential, AM encounters multiple obstacles that restrict its widespread adoption, including extensive pre and post-processing requirements, such as file preparation, cleaning, and finishing, which can be labor-intensive and add time and cost to production. Moreover, the reliance on skilled or semi-skilled labor increases production expenses and reduces scalability, making AM more suitable for low- to medium-production volumes. At higher scales, AM struggles with cost efficiency and consistency. Broader discussions to achieve industrial integration of AM require a focus on scalability, regulatory compliance, and sustainability. Sustainability, in particular, is critical in modern manufacturing, as alignment with global sustainability goals necessitates addressing energy consumption, carbon footprint, and lifecycle impacts.

Regulatory compliance is also essential for penetrating critical industries such as aerospace, medical, and automotive sectors, where standardization and trust are vital. Expanding AM’s applicability depends on leveraging emerging technologies, such as AI and machine learning (ML), which hold the potential to revolutionize it by optimizing design processes, predicting maintenance needs, and improving quality assurance. While AM excels in low-volume, high-complexity manufacturing, addressing the challenges of scalability is crucial for its broader adoption and competitiveness with traditional manufacturing methods.

By addressing these challenges and leveraging its strengths, AM can continue to evolve as a cornerstone of innovation in industrial processes, supporting advancements in sustainability, economic efficiency, and technological integration.

While the initial focus of AM was rapid prototyping, this technology’s inherent versatility has led to the multidisciplinary growth of AM, encompassing a wide array of applications. Below are two main fields where AM is employed [28].

- Aerospace Industry

Additive manufacturing has garnered attention in the aerospace industry due to its potential to reduce component weight, resulting in direct cost savings and design optimization. The need to achieve exceptional mechanical properties with the lightest weight possible has led to the use of expensive materials, such as superalloys, which are difficult to process using traditional methods, although they facilitate the creation and repair of metallic and non-metallic components for aerospace applications, such as aero engine parts or fixtures; the ability to reduce material waste and focus solely on obtaining high-quality raw material is highly attractive to this sector [28,29]. Additionally, AM facilitates the production of parts as needed, reducing inventory costs and maintenance time; this potentially transforms spare parts supply chains by facilitating localized production, minimizing the need for large inventories, and improving adaptability and resilience to disruptions. AM has demonstrated significant capabilities in the aerospace sector, from prototyping to end-use part production and repairs [30].

- Health Sector

Horn et al. noted that AM has been extensively explored in the healthcare field since this technology became accessible to the public. For example, in Europe during the 1990s, the Phidias project was developed, aiming to use rapid prototyping to generate models of patients’ anatomy and manipulate them in three dimensions. This has allowed doctors the opportunity to practice high-risk surgeries and gain a better understanding of the complex systems they work with.

Furthermore, additive manufacturing has contributed to the creation of customized medical instruments and orthopedic implants, offering the possibility of greater accessibility, as manufacturing them through traditional methods requires time and highly trained personnel. Another important factor is that the ability to manipulate geometries provided by this manufacturing method is highly useful in closely resembling human body parts, providing stability and strength with a reduced amount of material [28].

Environmental impact must be considered when proposing manufacturing alternatives. At first glance, it may be said that AM brings obvious benefits such as reduced material waste, decreased transportation needs, and improved energy efficiency compared to conventional manufacturing. However, this assertion cannot be made unequivocally.

Rejeski et al., in their research based on the 2024 National Science Foundation (NSF), highlight five key aspects for analyzing the environmental implications of AM: energy use, occupational health, waste management, lifecycle impact, and cross-cutting policy issues [31]. AM processes often consume less energy compared to traditional manufacturing methods due to their ability to directly produce complex parts without intermediate steps [31,32]. Additionally, AM’s capability to produce lightweight parts can lead to energy savings of up to 50% during the use phase of products such as machines, vehicles, or other systems. In the case of aeronautical components, transitioning to AM has been shown to achieve a 41.9% reduction in component weight, leading to substantial environmental advantages, such as decreased fuel consumption and lower CO_2_ emissions [32,33]. However, the environmental impact of energy use requires careful lifecycle analysis, particularly in feedstock preparation and machine operations, as these factors might offset the savings if not optimized [31]. The potential risks associated with AM processes include exposure to adverse environmental and occupational health impacts, such as fine metal powders, nanoparticles, and volatile organic compounds (VOCs). VOC emissions during AM, such as styrene, formaldehyde, acetone, and benzaldehyde, vary based on materials and processes. Styrene and formaldehyde have been identified as common emissions from ABS-based filament printing, with emissions influenced by printing temperatures and filament compositions [34,35]. Material extrusion printing releases VOCs at rates of 0.2–1.0 mg/h, with over 200 compounds detected, while stereolithography often exceeds 4 mg/h, producing higher VOC levels due to the thermal decomposition of resins [35,36]. These emissions contribute to poor indoor air quality and pose acute and chronic health risks, including respiratory irritation, cardiovascular disease, and cancer, particularly in inadequately ventilated environments [31,33,37]. The need for systematic risk assessment and management is crucial, including developing ’safer-by-design’ approaches and implementing them at the early stages of manufacturing processes. Suggested mitigation strategies include enclosing 3D printers, using high-efficiency particulate air (HEPA) filters, and adopting safer community practices to reduce exposure [31]. Waste management in AM processes is generally lower than those of conventional methods due to reduced material waste [32]; however, challenges persist in recycling materials, primarily due to compositional changes and property deterioration [31]. Efforts to reuse waste materials, such as repurposing them into filaments or powders for 3D printing, have been explored. An accurate generalization range of 60–98% of physical or chemical properties recovered from recycled AM materials depends on several factors, such as the type of material, the recycling method, the number of recycling cycles, and the processing conditions. Lower recovery percentages are more typical for polymers subjected to multiple recycling cycles without proper reconditioning or additives, and higher recovery percentages (~98%) are observed in metallic powders that undergo controlled reuse with proper sieving, oxidation management, and blending with virgin materials [38,39,40,41,42]. Additionally, the development of biodegradable or compostable filaments offers a promising avenue for sustainable practices, although issues such as low mechanical strength and poor moisture resistance remain significant obstacles [31]. The lack of a standardized framework to handle sustainability for AM processes, including discussed environmental implications, present challenges related to policy and legal liability [31,33]; these challenges are further intensified by the implementation of interdisciplinary manufacturing models, such as bioprinting and home-based production [31], reducing the environmental impact of AM and fostering a circular economy [32]. To mitigate these impacts, it is recommended to develop sustainability frameworks specifically tailored to the AM industry, enhance community practices for safer technology use, and establish proactive regulatory frameworks [29] to manage decentralized and emerging AM technologies [31,33].

AM technologies hold promise for reducing environmental footprints, addressing energy and material efficiency, waste management, and regulatory gaps as crucial for their sustainable adoption and for accurately quantifying the environmental impact generated. Specific timeline projections are limited; existing studies provide insights into potential scenarios based on Lifecycle Assessments (LCAs) comparing AM with conventional manufacturing (CM) and part consolidation (PC) to leverage the benefits of weight reduction, extended lifespan, and enhanced functional performance [43,44].

## 3. Methods

A literature review is undertaken in this section to examine the diverse methods presently used in additive manufacturing, seven categories of AM, namely, binder jetting, directed energy deposition, material extrusion, material jetting, powder bed fusion, sheet lamination, and vat photopolymerization, these have been recognized and defined in ISO/ASTM 52900 [1].

### 3.1. Binder Jetting (BJ)

Binder Jetting has been studied by MIT since the early 1900s; however, it is a non-beam additive technique not as extensively studied as powder bed fusion or directed energy deposition, but it has a wide range of potential improvements [5].

**Figure 2:**
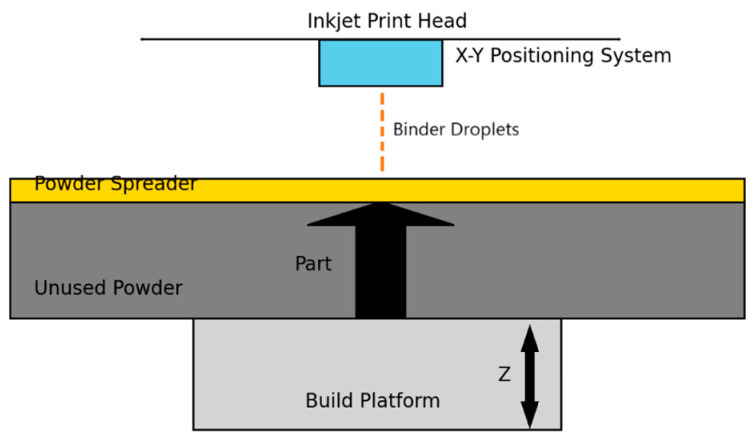
Schematic of binder jetting printing process.

The procedure is simple: a light layer of powder material is placed on the worktable, such as metals, ceramics, or polymers, Figure 2. Then, this layer is bound together by a liquid binder, usually a polymer, to give structure to each layer, applied only to specific parts [5,45]. This cycle continues until the component is fully formed. In essence, it mirrors the principles of 2D printing. Some of the advantages offered by this process include the surplus powder aiding in providing stability, thus reducing the need for support. Additionally, unlike other building processes, no heat source is involved, and it is conducted at room temperature, thereby reducing changes due to residual stress caused by temperature gradients [46].

One of the most commonly used metallic materials in this field is Stainless Steel 316L, which was studied by Mirzababaei. In their article, differences between components manufactured through AM and traditional manufacturing are mentioned, as well as the effects that temperature variations can entail. Parts manufactured through AM using SS 316L consist of γ-austenite and δ-ferrite, whereas in subtractive manufacturing, a complete austenitic phase is typically used. Due to the versatility of the material, various AM methods can be employed; however, it depends entirely on the application. BJ stands out for its viability in biomedical applications due to its internal porosity; however, this would be a drawback if superior mechanical properties were sought after [46].

Multiple factors determine the quality of the final product, such as powder size, materials, and post-processing. However, the general mechanical properties of the print are primarily influenced by the shape, size, and distribution of the raw material, as they affect the microstructure of the piece. Another significant characteristic of the powder is its flowability, which ensures consistent and uniform surface coverage. Typically, a larger powder size allows for proper particle distribution, while very fine powder tends to concentrate in specific areas and create voids in the bed, resulting in printing defects. It is important to strike a balance between these characteristics to achieve the desired results [46].

A drawback of this type of additive manufacturing is the need for post-processing to enhance mechanical properties. The most well-known post-processing method is sintering, which involves bonding particles using elevated temperatures without exceeding the melting point. This partial bonding process allows for the creation of three-dimensional objects with high precision and fine details as particles fuse rather than fully melt [46].

Different approaches exist in studying this manufacturing method. For instance, Ziaee conducted a study analyzing various powder preparations to achieve variable density. Using two types of SS 316L, one involving agglomerates of fine powder, while the other incorporated nylon 12 powder as a fugitive space holder to increase porosity, different materials can be used, but in this case, nylon was chosen because, in post-processing stages, it melts much faster than the base material. The research showed how the mixture’s spread density influenced final density post-sintering; it was observed that in materials without added nylon, the spread density greatly influenced the final density after the sintering process, with densities remaining at 92.2% and 93.9%, while samples with nylon behaved differently; initially, the mixture had high density, but during the sintering process, the nylon decomposed, resulting in larger voids than those naturally created by the BJ process; the final densities were 63.3% and 66.8% for mixtures containing 33% and 25% nylon, respectively. Porous parts can be used as filters, heat exchangers, and some other energy applications, demonstrating good resistance. This shows that it is possible to manipulate the natural porosity of this additive manufacturing method for our benefit [11].

The effect of different powder sizes and additives on achieving total density in pieces manufactured with SS 316L powder has been investigated by Rego et al. Four powder sizes and various boron compounds were utilized in the study. Through multiple tests, a specimen with a density of 98.13% was obtained by incorporating a 0.5% boron additive and sintering at 1300 °C. Densities of 99.67% and 98.33% were also reached with the addition of 0.75% of the additive. The mechanical properties of the sample were found to be comparable to those of bulk SS 316L, as the same level of hardness was achieved [47].

High-Speed Sintering (HSS) is an innovative additive manufacturing (AM) technology that combines aspects of powder bed fusion and binder jetting. It operates by inkjet deposition of a radiation-absorbing material in the desired pattern directly onto the powder surface, followed by infrared irradiation of the entire build area; the process then repeats until the build is complete [48].

Binder jetting is an excellent choice for applications where complexity, material variety, and speed are more critical than mechanical strength in the as-printed state; it is valued for its cost-effectiveness and ability to produce large components without heat-induced distortion. It is environmentally efficient, generating minimal waste. However, it has notable limitations, including shrinkage, high porosity, and reduced mechanical strength in printed parts, requiring extensive post-processing such as sintering or infiltration [49]. In principle, any polymer material available in powder form can be utilized in this process. Additionally, removal of support structure is relatively easy with this technique.

### 3.2. Directed Energy Deposition (DED)

**Figure 3:**
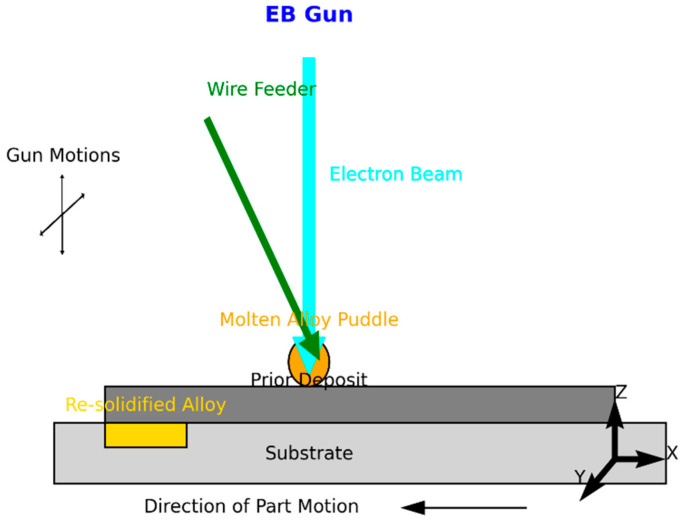
Schematic representation of wire electron beam process.

Directed energy deposition (DED) involves melting metallic material (wire or powder) with a heat source (laser, electron beam, or plasma arc), Figure 3; the process involves this heat source that generates intense thermal energy to melt the material due to their precision and ability to focus energy on small areas. Typically, a nozzle or similar mechanism, i.e., an Electron-Beam Gun (EB-Gun), delivers a wire or powdered form to the molten pool through Material Feeding System (MFS); the feed rate is carefully controlled to match the energy input and deposition speed. Upon cooling, the molten material solidifies and adheres to the substrate or the layers deposited earlier. The deposition head and substrate are moved in a controlled manner along multiple axes (usually three to five), enabling complex geometries and precise material placement. This process is repeated for successive layers and applied in the working area multiple times to create a tridimensional part. The mechanical properties in DED are achieved and tailored through careful manipulation of processing parameters, microstructural control, and material selection; internal properties, such as microstructure, can be customized by adjusting the material feed, energy input, and cooling rates [50]. However, employing these methods in metals such as titanium or aluminum, as well as their respective alloys, yields finishes that are not as sophisticated, necessitating post-processing and heat treatments to enhance their mechanical properties [51].

Potential in producing components with tailored mechanical properties has been demonstrated in aerospace and industrial applications; optimization of processing parameters and integration of advanced monitoring systems will further enhance the mechanical reliability and efficiency of DED-manufactured parts.

Ti6AL4V is a particularly attractive material due to its biocompatibility; nevertheless, when using AM techniques such as fusion-based, unsatisfactory results were obtained, especially in its porous and elongated microstructure; that is why today there are variations of the same method. Farabi et al. analyzed the micro-structure of Ti6Al4V alloys manufactured by a method patented by MELD Manufacturing Corporation, called additive friction stir deposition (AFSD); this is a deposition in the solid state since it uses other methods (friction in this case) to bond materials rather than affect their microstructure by melting them completely, this process improves their mechanical qualities as they resemble much more those of titanium alloys in traditional processes, even exceeding them, keeping a homogeneous microstructure [52].

During their study, they worked with Grade 5 Ti6Al4V; four different tests were conducted, where factors such as spindle speed, feed, and transverse rates were varied, along with analyzing the average temperature during the process to verify if it is a determining factor in specimen performance. ASTM E8-16a and STP 1576 standards were followed to prepare specimens for tension tests; researchers reported that samples fabricated at high deposition temperatures have yield strength of 913 and 875 MPa, with elongation values of 8.5% and 15.13%, respectively. Conversely, samples at lower deposition temperatures showed higher values, with 920 and 1010 MPa, along with elongation values of 15% and 18%. These results indicate a direct relationship between deposition temperature and the mechanical behavior of the material. Therefore, it is concluded that it is possible to manipulate the parameters to obtain uniform microstructures and thus achieve parts with properties comparable to, or even superior to, those manufactured using traditional methods.

Another way to optimize metal additive manufacturing is through the development of more sophisticated machinery. While a three-axis configuration was traditionally used for Laser Directed Energy Deposition (LDED), Kaji et al. highlight the benefits of employing a five-axis configuration. This allows the nozzle to remain tangent to the surface, reducing the need to build supports for creating angles. Unfortunately, this configuration increases the likelihood of collisions between the nozzle and the existing material. However, through simulation, it is possible to develop paths that avoid damaging the machines [53].

Direct energy deposition is a prominent technique known for its capability for rapid material application, achieving high deposition rates suitable for building large-scale components efficiently [30]. DED supports diverse materials, including metals, alloys, and composites, and enables the creation of intricate geometries without extensive reliance on support structures, particularly in advanced five-axis configurations [53]. Additionally, it excels in repairing and retrofitting components such as turbine blades, providing precise material addition, and reducing waste. Its ability to tailor microstructures allows for customized mechanical properties, while functional grading facilitates the production of components with gradient material properties optimized for complex environments. This innovative method also presents several challenges, as its limitations in surface finish issues often necessitate post-processing, increasing both time and cost. High thermal inputs can result in residual stress, distortion, and potential warping, complicating the dimensional accuracy and requiring effective thermal management strategies, and the reliance on skilled operators and high initial equipment costs limit its widespread adoption [51].

To conclude, DED offers significant benefits, including high deposition rates, material versatility, and effective repair capabilities, alongside challenges related to surface quality, thermal management, and operational complexity; it excels in industrial and aerospace applications where its unique strengths outweigh its disadvantages.

### 3.3. Material Extrusion (ME)

Fused Deposition Modeling (FDM), also known as material extrusion, was developed by Scott Crump, the co-founder of Stratasyn+ mkds, Ltd., in 1988. This additive manufacturing technique involves creating layers and mechanically extruding melted thermoplastic onto a build plate [5]. When molten filaments are deposited during material extrusion, it often leads to 3D-printed parts having direction-dependent properties, known as anisotropy [54]. While this additive manufacturing technique has gained popularity for home printing nowadays, there are initiatives to employ this technique with an industrial focus. It is a widely utilized additive manufacturing technique [55]. This method is notable for its cost-effectiveness, with other systems like Fused Filament Fabrication (FFF) being relatively inexpensive to assemble and operate.

ME offers several advantages, including support for a diverse range of materials, including commonly used polymers such as ABS and nylon, and environmentally friendly options like polyamide and Polylactic Acid (PLA), enabling broad application versatility [56]. Moreover, the process is characterized by the good mechanical integrity of produced parts, simple setup and operation, efficient creation of complex and customized designs, and reduced material waste due to precise deposition [57]. However, the method has limitations, including lower accuracy and surface finish quality compared to other additive manufacturing techniques. Additionally, thermal stress and warpage can impact dimensional stability, often necessitating post-processing to improve surface quality. The anisotropic properties of parts and constraints in nozzle design further limit their structural performance and complexity. Furthermore, the use of support structures can contribute to material wastage [57], although the use of a preheating platform and extruder is recommended for good adhesion which helps in reducing failures and fabrication costs. Multiple prints of a Hilbert cube, varying the slicing technique and the type of support used, were studied by Ghais Kharmanda; generally, the software provides two types of support for printing overhangs: linear and tree-like. Linear supports are vertical structures protruding from the object, resembling pillars, and are simple and effective. On the other hand, tree-like supports feature branched geometry, typically ideal for complex geometries or irregular shapes due to their significantly lower material usage and easier removal. Given the small and cubic geometry in this study, linear supports were generally more effective for printing, and to avoid affecting the stability of AM process, a recommendation was made to apply preheating to platform for good adhesion, which helps in reducing failures and fabrication costs [57].

### 3.4. Material Jetting (MJ)

**Figure 4:**
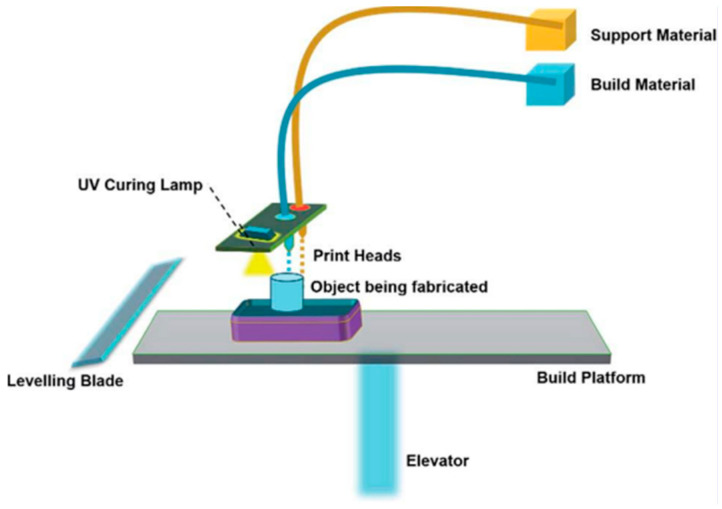
Schematic representation of material jetting [58].

Like many additive manufacturing techniques, MJ varies in its configuration depending on the manufacturing purpose; however, a general outline of this process is illustrated in Figure 4. Broadly speaking, a print head deposits tiny droplets of material by utilizing piezoelectric or thermal mechanisms to eject these droplets at precise locations on the build platform. The materials are stored in separate reservoirs for both build and support materials. The first layer on the platform is cured using a UV curing lamp, which solidifies the liquid photopolymer into a solid state, ensuring strong bonding between layers. After each layer is formed, the build platform descends by a set amount, allowing room for the next layer. This cycle continues until the complete 3D object is constructed. The leveling blade ensures that each layer of material is evenly distributed before the next layer is deposited, maintaining uniform layer thickness and dimensional accuracy. To generate support structures, a separate material is used for overhanging parts of the object. These supports are necessary to stabilize the geometry during printing. Post-processing involves removing these support structures using mechanical or chemical methods. This process allows for high resolution and excellent surface finish due to the precision of the print heads, and thin layers can be produced as small as 16 microns. An example of a curing method is ultraviolet (UV) light, which is utilized at wavelengths between 190 and 400 nm. Since MJ is a layer-by-layer manufacturing method, support material is used for overhangs, which are removed upon completion of the print. It is a highly versatile procedure that allows for the combination of different materials and colors, with the purpose of enhancing mechanical properties and fabricating complex multi-material parts. This versatility makes MJ stand out for the manufacturing of composite materials [58].

Sugavaneswaran et al. propose a manufacturing technique aimed at improving the strength of a material through the application of random oriented multi-material (ROMM) [56]. For this purpose, samples of parts with pure elastomer and elastomer reinforced with fibers were produced using a 3D printing machine. To add reinforcement, CAD software CATIA VB SCRIPT was utilized, where an algorithm was generated that receives as input the volumetric percentage and aspect ratio of the fiber, which were declared as 10 Vol% and 1:10, respectively, in the tests. Then, parameters delimiting the work area are entered, and the algorithm is responsible for generating the random reinforcement pattern. Tensile tests are used to characterize the samples and, thus, analyze the mechanical properties obtained, always following standardized standards, such as ASTM. In some case studies, it has been concluded that reinforcements increased stiffness by 22% and elongation by 10% compared to pure elastomer elements. This research demonstrates the potential of ROMM components fabricated via Polyjet 3DP to enhance part performance and versatility; however, challenges such as stress–strain inconsistencies cause irregularities at low elongations, reinforcement de-bonding under tensile loads initiated cracks leading to unpredictable stress concentrations and fracture, and at last, material limitations made parts weaker restricting the use in high-stress applications [59].

MJ is an advanced AM technique recognized for its excellence in producing intricate and visually appealing components. One of its most notable advantages is its multi-material capability, allowing simultaneous deposition of different materials or colors, making it ideal for creating complex gradients in color, transparency, and stiffness [60]. The process also delivers high precision and fine resolution, making it suitable for intricate designs while producing components with a smooth surface finish that surpasses many other AM methods. MJ excels in manufacturing functional prototypes with detailed features and ensures homogeneous material properties throughout the printed parts. In spite of the challenges, the technology has notable limitations. It is primarily restricted to photopolymers and waxes that can form droplets, limiting its material versatility and applications. The high cost of these materials elevates production expenses and often generates significant material waste, particularly from the support structures required for complex geometries. MJ also faces size constraints, making it less suitable for manufacturing large parts [55].

### 3.5. Power Bed Fusion (PBF)

In this technique, in which a heat source of considerable energy (such as a laser or electron beam) selectively melts a fine layer of powdered material, typically metals or polymers to build components layer by layer [50]. Subsequently, the next layer is deposited using a rolling mechanism and brought to the melting point to fuse with the previous layer. Within the PBF family, there are different techniques, such as Electron Beam Melting (EBM) and Selective Laser Melting (SLM) for metals and Selective Laser Sintering (SLS) for polymers. Among these, SLM is one of the most commonly employed techniques for metal alloys due to its ability to fully melt the material, ensuring high-density components. SLM stands out for its precision compared to other additive manufacturing techniques like directed energy deposition. However, it requires very high temperatures within specific ranges to function properly, as the energy input in the SLM process must be precisely controlled, as excessive energy can cause material evaporation or defects such as keyholing, whereas insufficient energy may lead to incomplete fusion and porosity formation [61]. This poses particular challenges when working with polymers, as temperature variations can affect their lifespan [5] and rely heavily on precise energy input parameters to achieve desired material properties [62].

To ensure the reliability of the results, stable parameters were determined within the experiment, including a layer thickness of 100 μm, powder application speed of 250 mm/s, hatch distance of 250 μm, and a building chamber temperature of 172 °C. Additionally, the following equation was used to define the resulting energy density (*E_D_*):

(1) $$E_{D}=\frac{P_{L}}{V_{s}h_{s}d}\;$$

where the variables laser power (*P_L_*), scan speed (*V_S_*), hatch distance (*h_S_*), and layer thickness (*d*) were utilized [62]; energy density (*E_D_*) is determined by these factors that directly influence material consolidation, component density, and mechanical integrity. As highlighted in the literature, achieving the optimal energy density is critical for minimizing defects such as porosity, thermal degradation, and poor interlayer bonding, which can compromise component performance. Samples with variations in laser power and scan speed were prepared as the previous literature indicated that these were the factors directly impacting polymer aging. After preparing the required specimens, tensile testing was performed to evaluate their mechanical properties. The results demonstrate that as energy density or laser power increases, the component density remains constant within the measurement precision. However, a reduction in scan speed increases the porosity of the component, as the polymer does not fully melt, thereby reducing its density. It was also determined that an energy density of 0.35 J/mm^3^ optimizes the component density.

Researchers aim to refine energy density parameters to improve reproducibility and extend the application in diverse industries. PBF is a highly advanced AM technique for its ability to create intricate and complex geometries, it offers high resolution and accuracy, particularly in processes like Selective Laser Sintering (SLS) and SLM, making it suitable for applications demanding fine detail. The technique efficiently utilizes space in the build chamber through part nesting, allowing multiple parts to be manufactured simultaneously.

Although metal PBF-printed components offer significant advantages, they often necessitate extensive post-processing to improve their mechanical properties and dimensional precision. One of the primary steps is support removal, as many PBF-manufactured metal parts necessitate support structures during printing to prevent warping and distortion. These supports are manually or chemically removed, followed by surface finishing to eliminate residual roughness [63]. On SLM, the support removal is critical due to the high thermal gradients that lead to significant residual stress, often requiring additional machining or electrochemical polishing [9], and EBM supports are generally easier to remove because the process occurs in a vacuum, reducing thermal stress and minimizing bonding between supports and the part [64]. SLS typically eliminates the need for support structures since the encompassing powder naturally supports the printed layers throughout the process [61].

Additionally, heat treatment is crucial due to the residual stress induced by rapid melting and solidification cycles. Stress-relief annealing helps mitigate internal stresses, preventing cracking and improving ductility, while hot isostatic pressing (HIP) is particularly valuable in aerospace and biomedical applications for eliminating porosity and enhancing fatigue resistance [8,65]. Extensive heat treatment to relieve stresses on SLM enhances mechanical properties and prevents part failure. HIP is often employed to improve density and fatigue strength [66]. For EBM, heat treatment is less critical than in SLM, as the process inherently has lower residual stresses due to slower cooling rates and vacuum conditions [67]. Treatment is mainly used on SLS for polymer parts rather than metal components, though post-sintering steps can improve densification [68].

Another essential step is surface finishing, as the layer-by-layer nature of PBF results in high surface roughness, impacting mechanical behavior and wear resistance. Techniques such as shot peening, machining, polishing, and chemical etching are commonly applied to improve the final part quality [69]. Surface roughness on SLM parts is a significant issue, often necessitating CNC machining or electrochemical polishing to achieve smooth surfaces suitable for aerospace and medical applications [70]. EBM produces smoother surfaces than SLM but still benefits from post-processing techniques such as abrasive blasting and chemical etching [65]. On SLS polymer parts, vapor smoothing or bead blasting is used to improve surface quality [71].

Finally, mechanical testing and quality control are conducted to ensure the printed components meet industry standards. These evaluations include microstructural analysis, tensile testing, and fatigue analysis, verifying the reliability and performance of the final product. SLM requires extensive mechanical testing to confirm density, residual stress relief, and mechanical integrity due to its high-energy laser process [72]. Typically, EBM shows lower residual stress, but microstructural analysis is essential to verify uniformity [73], and SLS is primarily evaluated for dimensional accuracy of polymer-based parts [74].

Regardless of these post-processing obstacles, PBF remains a versatile and robust manufacturing method, especially for producing high-quality parts.

### 3.6. Sheet Lamination (SL)

**Figure 5:**
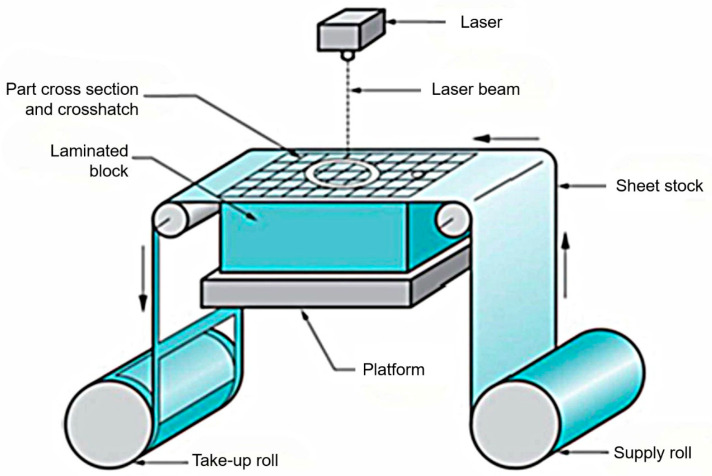
LOM—Laminated Object Manufacturing process [75].

SL is a type of Additive Manufacturing where metal sheets are bonded together to create a solid object [1]. This process is often recognized for its hybrid characteristics, combining additive and subtractive methods. SL is widely used due to its capability to process various materials like polymers, paper, and metals while maintaining lower costs compared to other AM methods [75,76,77,78]. Two primary variants of SL are Ultrasonic Consolidation (UC) and Laminated Object Manufacturing (LOM). The LOM system was patented in 1987 by the company formerly known as Helisys Inc. (now Cubic Technologies) [5]. As illustrated in Figure 5, the LOM method is characterized by the successive gluing of laminates, typically comprised of paper or plastic, which are cut to the desired shape through a laser cutting process. The workflow begins with a sheet being joined to a substrate via a heated roller, after which a laser precisely delineates the model’s dimensions by cutting away non-essential areas. Upon completing a layer, the platform is lowered to accommodate the next sheet, and the procedure is repeated until the final object is built [75].

This prototyping technique offers versatility, functioning on multiple substrates that can embody adhesive-coated paper and thin metal sheets. Although LOM shares certain similarities with lithography and other sheet processes, it uniquely integrates laser technology to define object geometries. A significant advantage of this method is its ability to produce solid physical models efficiently, with the option for post-processing modifications such as machining or drilling to enhance the final product.

Additionally, studies have been conducted to enhance manufacturing finishing besides the use of metals, an example of which is the study by Liao et al., where they proposed the use of self-adhesive sheets to avoid errors generated when trimming the excess material before considering the final layer position. This support system demonstrated a reduction in manufacturing time as well as between 30% and 80% of material waste, and it increased the possibility of creating more complex geometries with better finishes [79].

Some of the advantages offered by this type of additive manufacturing include the opportunity to combine different types of metal sheets with significant precision and a straightforward process [80]. Additionally, the review highlights advancements in related techniques, such as Ultrasonic Additive Manufacturing (UAM), which has gained attraction for its effectiveness in stacking metal layers with minimal thermal distortion, ideal for creating gradient scaffolds used in tissue engineering [81]. LOM’s composite capabilities extend to embedding electronics within objects, giving it an edge in sectors requiring intricate designs paired with added functionalities [82].

### 3.7. Vat Polymerization (VPP)

This AM method provides the possibility of high-quality finishing and good property control at relatively low cost thanks to the ability to detail the model on a microscopic scale. The process consists of layer-by-layer curing a liquid photopolymer resin (monomers, oligomers, photoinitiators) through a light or laser source (generally UV light) on a work platform [83]. The VPP processes include Stereolithography (SLA), Digital Light Processing (DLP), Continuous Digital Light Projection (CDLP) and Two-Photon Polymerization (2PP). For clarity and conciseness, this discussion will address SLA and DLP.

**Figure 6:**
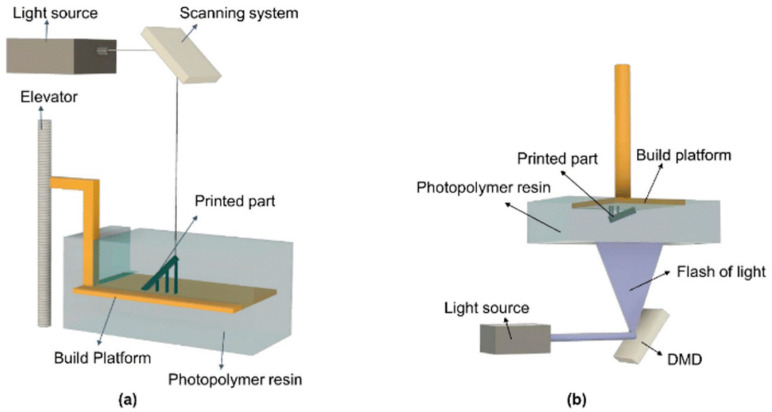
Schematic illustration of (**a**) the top-down approach using vector scanning and (**b**) bottom-up approach utilizing mask projection [84].

Refer to Figure 6; the VPP printing process can be classified according to platform motion and the type of light exposure used. The platform has two main configurations: top-down, where the platform moves downwards, and down-top, where the platform moves upwards [84]. The most commonly used configuration in the industry is the top-down because it reduces the possibility of part failure during printing.

In the top-down method, Figure 6a, the build platform starts above the resin and moves downward, equal to the layer thickness after each exposure to light, while a recoated blade spreads fresh resin for curing. This process continues until the entire 3D object is fully formed. In the bottom-up approach, Figure 6b, the build platform starts at the bottom of a transparent resin vat. The light source cures the resin from below, and the platform moves upward to allow fresh resin to flow in. This process repeats until the final 3D part is fully constructed.

An important approach for VPP is the creation of Functionally Graded Material components. Nohut et al. define them as a new class of compounds with continuous variation in composition and microstructure throughout the entire volume that provides exceptional properties. This change in their microstructure distinguishes them from the individual materials that make them up. Some of the most important technologies in VPP for Functionally Graded Materials are Stereolithography and DLP.

SLA achieves fine details through laser-scanning technology, while DLP utilizes a digital micromirror device (DMD) to cure entire layers simultaneously. SLA has become integral in industries such as aerospace, automotive, and biomedical engineering due to its capability to produce complex geometries with superior surface finish and dimensional accuracy [83,84]. This technology relies on a UV laser to selectively cure liquid photopolymer resin, enabling the creation of intricate designs with excellent detail resolution. SLA offers advantages such as rapid prototyping, customization, and compatibility with diverse photopolymer formulations, making it ideal for applications requiring high precision. Advancements have focused on improving material properties, increasing printing speed, and integrating AI-driven automation to enhance process efficiency [85]. However, challenges remain, including the brittleness of printed parts and post-processing requirements. To address these challenges, dual-curing polymer systems integrate SLA with secondary thermal curing, enhancing toughness, durability, and structural integrity [86]. This hybrid approach allows for improved crosslinking density and interfacial adhesion, making SLA more viable for applications in biomedicine, soft robotics, and high-performance functional materials.

Additional research explores how variations in curing duration and temperature influence the mechanical characteristics of resins produced via SLA printing [87]. Optimal post-curing conditions were determined to be 90 min at 60 °C, improving tensile strength and stiffness. However, exceeding these limits led to performance degradation. These findings underscore the importance of accurate post-curing parameters in enhancing the durability of SLA resin.

Research has focused on enhancing SLA resins with nanofillers such as graphene, carbon nanotubes, and metal nanoparticles, significantly improving mechanical strength, electrical conductivity, and thermal stability [88]. Advances in SLA-based nanocomposite formulations aim to optimize polymerization kinetics, nanoparticle dispersion, and curing parameters to enhance material properties. Despite these improvements, challenges remain in resin recyclability and process sustainability. Future developments should focus on expanding the functionality of SLA materials while addressing environmental concerns and improving process efficiency.

For specialized applications, studies have validated the SLA-based fabrication of microchannels for capillary-driven fluid flow, highlighting the impact of geometry, surface roughness, and manufacturing parameters on flow behavior. The findings demonstrate stable fluid velocity in channels ranging from 300 to 800 µm, confirming SLA’s feasibility for microfluidic applications. Additionally, an optimized printing process incorporating an air-cleaning step enhances accuracy and improves the reliability of SLA-printed microstructures, supporting the advancement of cost-effective lab-on-a-chip systems and biomedical diagnostics that require precise fluid control [89].

Despite these benefits, as previously indicated, both techniques have notable limitations. The systems and photopolymer resins are costly [55], and the resins require careful handling due to their toxicity [84], with post-processing steps like cleaning and curing adding to the time and complexity. The printed parts often lack the mechanical strength needed for functional, load-bearing applications, and the techniques are generally limited to producing small-to-medium-sized objects due to constraints in the light source and vat tank size [90].

### 3.8. Four-Dimensional Printing (4DP)

Four-dimensional printing builds upon 3D printing by introducing the time dimension, allowing printed structures to adapt their shape or function when exposed to external stimuli such as light, heat, moisture, or magnetic fields. Unlike conventional 3D printing, which produces static objects, 4DP utilizes smart materials with shape-memory properties or stimuli-responsive characteristics, enabling dynamic transformations. This technology builds upon additive manufacturing techniques like vat photopolymerization, material extrusion, and powder bed fusion, integrating programmable materials that react to environmental changes. The key advantage of 4DP lies in its ability to produce adaptive structures with applications across various industries. In aerospace, 4DP components can self-adjust for optimal aerodynamic performance, reducing weight and enhancing fuel efficiency [91].

Research highlights its potential in energy storage, impact resistance, and self-folding structures. Efforts focus on refining material properties, durability, and multi-material integration to enhance industrial viability, positioning 4D printing as a key technology for next-generation smart textiles and engineering solutions [92]. Four-demsional ceramic printing is highlighted as a significant development, including shape memory ceramics, elastomer-derived ceramics, and additive–subtractive manufacturing techniques.

Shape memory ceramics introduce tunable mechanical responses under external stimuli, making them suitable for aerospace, biomedical, and structural applications. Elastomer-derived ceramic 4D printing expands design flexibility, enabling structures with morphing capabilities [93]. Shape-memory polymers (SMPs), composites, and smart alloys have been studied. SMPs demonstrate efficient shape recovery when exposed to external triggers such as heat, light, or humidity. Composites, particularly those integrating fiber reinforcements or hydrogels, enhance mechanical strength and responsiveness, improving deformation control. Shape-memory alloys (SMAs) offer superior mechanical properties and repeatability but require precise thermal activation and optimized printing parameters [94,95].

A strong emphasis is placed on biomedical applications, including drug delivery systems, orthopedics, tissue engineering, and medical devices, where 4D-printed structures demonstrate enhanced functionality for minimally invasive surgeries and customized implants [96].

The primary challenges of this technology revolve around material selection, process control, scalability, and application feasibility. A critical issue is the limited availability of stimuli-responsive materials with consistent mechanical properties, biocompatibility, and long-term stability. Shape memory polymers, liquid crystal elastomers, and smart alloys require extensive optimization to enhance their programmability, recovery behavior, and durability under repeated activation cycles. Another challenge is precise control over actuation mechanisms, where achieving uniform stimuli response (thermal, light, pH, magnetic, or moisture-driven transformations) demands advanced computational modeling and real-time monitoring to predict deformation accurately. Scalability and manufacturing resolution remain obstacles, as current 4D printing methods, particularly vat photopolymerization and extrusion-based techniques, struggle to produce high-resolution, defect-free structures at an industrial scale. Multi-material printing and hybrid fabrication strategies are being explored to improve structural complexity and functional integration, but ensuring repeatability and cost efficiency is still a challenge.

To effectively visualize the different 3D printing methods [1], the following comparative information in Table 1 has been implemented for presenting a summary comparison of these seven AM techniques of AM: BJ, DED, ME, MJ, PBF, SL, and VP, along with their applications, benefits, and drawbacks and so on.

## 4. Materials Used to Fabricate with Additive Manufacturing

AM utilizes a broad selection of materials chosen according to specific application requirements. These materials are integrated into the design strategy to optimize mass, minimize waste, and enable the production of complex geometry challenges that traditional manufacturing methods struggle to address due to time-consuming toolmaking and post-processing.

A thorough understanding of AM requires knowledge of its materials [56]. The most commonly used categories in 3D printing include metals, polymers, composites, ceramics, and, more recently, smart materials. These materials come in different forms, including solids, liquids, powders, sheets, wires, and slurries. Their properties, including mechanical, thermal, electrical, chemical, and optical characteristics, differ according to the application and specific requirements.

### 4.1. Metals and Alloys

Metal 3D printing is experiencing significant growth. According to the Wohler Report, the number of companies selling AM systems for metal parts increased by 27.2% in 2023. This market has been tracked for 20 years, and sales data show a notable expansion, with 3049 metal AM systems sold in 2022 compared to 2397 in 2021, reflecting an 18.3% global industry growth [102].

Srivastava et al. identified Directed Energy Deposition (DED) and Powder Bed Fusion (PBF) as the two main commercial systems for metal 3D printing. Newly developing methods, including cold spraying, friction stir welding, binder jetting, and direct metal writing, are gaining popularity due to their higher accuracy and speed, and their industrial applications are expanding. Metal 3D printing involves the use of metallic feedstock, either as powder or wire, which is melted layer by layer using energy sources like lasers or electron beams [56]. A variety of metallic powders have been created, particularly stainless steels 316 and tool steels as maraging steel or H13; aluminum alloys AlSi10Mg, Al-Mg, or Al-Cu-Mg for aerospace applications; titanium alloys Ti-6Al-4V for prosthetics; nickel- and cobalt-based alloys Co-Cr-Ni or Inconel 718 for gas turbine engine components; copper alloys C-18150 for cooling fins and heat transfer systems; and rare-earth metal alloys for jewelry. These are highly compatible with the AM process [30].

Other studies examine Ni–Ti Gyroid lattice structures fabricated via LPBF [103], demonstrating that increasing interlacing cells enhances hyperelastic recovery and reduces residual strain, making them ideal for biomedical applications. While complexity affects manufacturability, stress distribution improves, preventing localized failure. Experimental and simulation results confirm superior mechanical performance, with recoverable strain exceeding 98%. These structures offer high elasticity, resilience, and tunable mechanical properties for applications like bone implants and aerospace components. To better organize the classification of metals and alloys according to their key applications and mechanical properties, Table 2 provides a structured overview that facilitates an understanding of their industrial and practical relevance.

#### 4.1.1. Steels

Steels are the most widely used metals worldwide, making them indispensable in various industries [104]. Different types of steel are commonly processed using PBF-laser and DED-laser AM techniques [9,30].

Austenitic stainless steel, particularly AISI 316L, is the most utilized in PBF-laser systems. This alloy, exclusively distributed by AM system manufacturers, is widely chosen for industrial applications due to its unique microstructure and high performance in structural components, mainly because of its corrosion resistance [105]. However, another austenitic alloy, 304L stainless steel, has gained attention due to its susceptibility to increased porosity and cracking, which reduce strength and ductility when fabricated via selective laser melting method [106,107]. Precipitation-hardenable steels, such as 17-4 PH martensitic stainless steel, offer high strength and good corrosion resistance, making them suitable for applications exposed to temperatures above 315 °C. This alloy is well-suited for AM, particularly laser powder-bed fusion, due to its good printability. Its microstructure strongly depends on processing conditions, as retained austenite is present after printing. However, solution annealing and aging effectively break up dendritic solidification, resulting in a microstructure containing 90% martensite and 10% retained austenite [108]. Another precipitation-hardenable alloy, 15-5 PH stainless steel, is similar to 17-4 PH but less commonly used. AM studies comparing PBF-laser and conventional processing have shown that AM-produced 15-5 PH exhibits shorter and narrower martensitic laths and higher microhardness than wrought material [109]. Both austenitic and precipitation-hardenable stainless steels are highly sensitive to AM parameters, requiring precise control to ensure optimal properties.

#### 4.1.2. Aluminum Alloys

Aluminum Alloys (Al) are widely used in engineering due to their strength, corrosion resistance, machinability, and cost-effectiveness, making them ideal for industrial applications. However, SLM of aluminum alloys presents unique challenges due to their material characteristics. The most used alloys in AM are AlSi10Mg and AlSi12, both valued for their mechanical properties [9,110]. Despite these advantages, processing aluminum via SLM is more complex than materials like stainless steel or titanium. This is mainly due to aluminum’s high laser reflectivity, which reduces melting efficiency. Additionally, its high thermal conductivity rapidly transfers heat away from the melt pool, causing uneven melting and solidification. Oxide layer formation on the melt pool surface further hinders the process, increasing the risk of defects [111,112]. The low viscosity of molten aluminum limits the size of the melt pool, making laser-PBF preferable over DED for aluminum manufacturing [30].

Research has focused on improving aluminum processing in AM, particularly for AlSi10Mg. Studies by Thijis et al. show that high thermal gradients during SLM create an extremely fine microstructure in AlSi10Mg, leading to high hardness even without aging treatment. Anwar and Pham found that optimizing scanning patterns against the gas flow and increasing inert gas velocity enhances ultimate tensile strength (UTS). This effect occurs because spattered powder accumulates near the outlet instead of burning in the laser beam, improving part quality. Achieving high-density aluminum parts requires careful parameter optimization, including higher laser power, to overcome SLM challenges and ensure high-quality production.

#### 4.1.3. Titanium Alloys

Titanium alloys are widely used, particularly in aerospace and biomedical applications, due to their high tensile strength and toughness. However, traditional processing methods are complex and costly, limiting their broader adoption [9].

Ti-6Al-4V is the titanium alloy most frequently used in AM, often requiring post-processing heat treatments to reduce residual stress. Its suitability for AM comes from its dual-phase (α + β) microstructure, which can be controlled to enhance strength but may also affect ductility and fatigue performance [9]. A key challenge in printing Ti-6Al-4V is managing defects such as porosity caused by inconsistent melt flow, rapid solidification, and powder layering issues, including high scanning speeds or thick powder layers [12,113].

The mechanical properties of Ti-6Al-4V in metallic 3D printing depend on post-processing conditions such as heat treatment, hot isostatic pressing (HIP), and annealing. According to Herzog et al., its yield strength typically ranges between 800 and 900 MPa, while its ultimate tensile strength (UTS) spans 950–1100 MPa, varying based on the additive manufacturing method used (e.g., SLM, EBM). Post-processing improves mechanical performance by refining the microstructure, reducing residual porosity, and mitigating stress concentrations, which enhances fracture toughness. Heat treatments and HIP further optimize yield strength and UTS by eliminating crack-initiating defects.

Ti-6Al-4V is gaining prominence due to its design flexibility and potential for weight optimization, making it a cost-effective solution for complex structures with minimal waste. It is also widely recognized for its biocompatibility. Qui et al. have explored non-stochastic cellular structures, such as rhombic dodecahedrons and trabecular designs, to create lightweight yet structurally resilient components. These designs are particularly well-suited for aerospace and biomedical applications, as they can mimic bone’s mechanical properties and reduce stress shielding [114]. Cellular structures, especially trabecular-like configurations, optimize energy absorption under load, while non-stochastic designs provide precise control over porosity, balancing structural rigidity and lightweight characteristics.

#### 4.1.4. Nickel- and Cobalt-Based Alloys

Inconel 625 and Inconel 718 are high-performance nickel-based superalloys widely used in AM, particularly in processes like SLM and Electron Beam Melting (EBM) [9]. Inconel 625 has a YS of approximately 500–600 MPa and a UTS of ~900 MPa, while Inconel 718 exhibits higher strength, with a YS of ~900 MPa and a UTS of ~1200 MPa [9]. Their strong resistance to thermal stress and excellent mechanical properties make them ideal for aerospace and high-stress environments. However, optimizing their performance requires careful control of the transition from columnar to equiaxed grain structures (CET) and post-processing [113,115]. Inconel 718 achieves higher strength through precipitation hardening, while Inconel 625 relies on solid solution strengthening from molybdenum and niobium. Their mechanical properties, including high strength, corrosion resistance, and thermal stability, can be further improved by optimizing grain structures, refining surface textures, and controlling hierarchical porosity to enhance isotropy, ductility, and fatigue resistance.

Incorporating bioinspired structures into AM could drive the development of advanced designs by mimicking natural patterns within the metal matrix. This approach could enhance grain structure optimization, surface texturing, and hierarchical porosity. The flexibility of AM enables precise control of these features, layer by layer, through adjustments in laser parameters such as power, scan speed, and hatch spacing, allowing for the tailored production of high-performance components.

#### 4.1.5. Copper Alloys

Copper alloys are highly valued in additive manufacturing for their excellent thermal and electrical conductivity, making them ideal for heat exchangers, electrical contacts, and high-performance cooling systems [30,56]. However, their high reflectivity and thermal conductivity complicate laser-based additive manufacturing, particularly in laser powder bed fusion, requiring precise parameter optimization to ensure effective melting and layer bonding while maintaining process efficiency and part quality [116].

When processed correctly, pure copper exhibits excellent ductility and strength. However, its anisotropic properties, resulting from layer-by-layer manufacturing, require careful control of scanning strategies and thermal gradients. Process optimization allows for tailoring mechanical properties, ensuring structural stability and prolonged durability in extreme conditions. Additionally, copper’s natural corrosion resistance is particularly beneficial for medical implants, where its antimicrobial properties help reduce infection risks. Nonetheless, as-built additive manufacturing parts often present surface roughness and porosity, which can compromise corrosion resistance [117]. Post-processing methods, including heat treatments, refine the microstructure and reduce residual stresses, while surface finishing techniques improve smoothness and minimize defects.

Copper alloys are ideal for fabricating bioinspired structures, such as honeycomb designs, which mimic natural load-bearing or flow-optimized geometries to enhance strength-to-weight ratios. These structures can be tailored for specific mechanical and thermal applications [118]. Additionally, copper alloys are frequently used in multi-material manufacturing, often paired with high-strength alloys like maraging steel, which have been successfully fabricated using additive manufacturing [116]. To fully exploit their potential, an integrated approach is required to address material, mechanical, and surface challenges.

### 4.2. Polymers

Polymers are widely used in additive manufacturing due to their versatility, affordability, and ability to create complex geometries. Key advantages include design freedom, cost-efficient prototyping, and accessibility in various forms, such as thermoplastic filaments, reactive monomers, resins, and powders [30,56]. Common methods include photopolymerization, material extrusion, and material jetting, with thermoplastics and UV-curable polymers being the most frequently employed [16]. Examples such as polyamide, PLA, ABS, and nylon are highly compatible with these processes [119].

A major challenge in polymer-based additive manufacturing is material wastage, particularly in complex models with overhangs and poorly positioned supports.

Ghais Kharmanda’s research on PLA explored temperature variations between 190 °C and 220 °C to enhance its mechanical and structural performance. The study used filaments derived from corn starch or sugar cane for their eco-friendliness and concluded that preheating conditions significantly impact print stability, especially in the early stages. While some suppliers recommend adhesives to improve initial print adhesion, excessive use may damage the platform when removed. Experimental results suggest an optimal extruder temperature of 240 °C and a platform temperature of 100 °C [57].

The HSS technique is faster than SLS and produces stronger parts by fusing powder rather than bonding it [48]. Ellis et al. studied Nylon 12, the standard material for HSS, and identified a linear relationship between print density and crystallinity, showing that as print density increased, crystallinity decreased. Mechanical testing revealed that higher crystallinity improved stiffness and tensile strength but reduced ductility. Drummer et al. analyzed how energy density in the selective laser melting (SLM) process affects material properties [62], particularly in polyamide 12, which is often sensitive to degradation at high temperatures. Polyamide 12 was chosen due to its well-documented powder flow behavior, low melt viscosity, and significant difference between melting and crystallization temperatures.

Despite decades of development, SLS still faces challenges such as limited material availability, anisotropic properties, and mechanical strength deficiencies [120]. Vazquez et al., in their research, utilized commercially available polyamide powders (PA-11 and PA-12) and proposed characterization methods to optimize the process. However, the range of suitable materials remains restricted, leading to ongoing research into elastomers and composite polymers.

Material extrusion-based additive manufacturing relies on melting and solidifying thermoplastic materials layer by layer [26]. FDM is a popular technique because of its fast processing and cost efficiency. Many FDM printers incorporate dual-nozzle systems for multi-material printing and are compatible with standard thermoplastics such as ABS, PLA, and PETG, as well as engineering-grade materials like polyamide and thermoplastic polyurethane. High-performance thermoplastics like polyether ether ketone and polyetherimide are also widely used. Unlike SLS and HSS, which form chemical bonds through cross-linking to produce fully dense parts, FDM primarily relies on mechanical bonding between layers [9].

PBF techniques employ UV-curable polymers, where monomers undergo selective polymerization in a resin tank activated by a photo-initiator and a light source [56]. Photopolymer-based systems offer exceptional accuracy, thin layer deposition, and fine detail precision. However, optimizing resin viscosity at low temperatures remains a challenge for PLA. This issue can be mitigated by increasing processing temperatures or incorporating plasticizers to reduce the risk of thermal degradation [16]. Despite advancements, further improvements in photopolymers’ thermomechanical properties are needed to expand their applications.

### 4.3. Composites

Composites are an advanced class of materials that emerged later than polymers and metals [56]. They are designed by combining two or more different components to improve properties beyond those of the individual materials. In 3D printing, PLA and ABS are among the primary polymers used for composite fabrication [30]. The incorporation of fibers or particles into polymers and metals significantly improves mechanical properties, making composites valuable in aerospace and sports applications. Various additive manufacturing techniques, including SLA, SLS, FDM, 3D bioprinting, and inkjet printing, can be used to produce composites [26], with FDM being the most widely adopted due to its accessibility [119].

Research by Brancewicz-Steinmetz et al. highlights the potential of layered printing techniques and filament modifications to enhance PLA properties, addressing the need to reduce dependence on petrochemical resources. Laminar composites, extensively used in multiple industries, can be efficiently fabricated via 3D printing. Optimizing mechanical properties requires precise control of manufacturing parameters, such as low printing speed, reduced layer height, and an appropriate reinforcement material ratio. Strength tests on ABS and PLA composites reinforced with carbon fiber indicate that multi-material samples exhibit superior strength compared to single-material counterparts [16].

Polymer matrix composites in 3D printing hold significant potential for industrial applications, offering exceptional functionality and mechanical performance. However, challenges remain, particularly regarding the limited range of printable materials suitable for high-performance composites and the need for process adaptations. Additionally, the speed and repeatability of additive manufacturing for composites are still inferior to traditional methods. Nevertheless, the shift from rapid prototyping to mass customization has accelerated efforts to develop new matrix materials with enhanced mechanical properties.

Recent advances in fiber-reinforced composites using FDM have greatly improved the mechanical properties of 3D-printed components. However, key challenges persist, including fiber orientation, adhesion between fiber and matrix, and void formation, all of which affect performance [30]. Further research is needed to expand material options and applications for 3D-printed polymer composites.

For instance, Rajakaruna et al. used an innovative method by combining PLA with hexadecyltrimethoxysilane (HDTMS) and polytetrafluoroethylene (PTFE) using solvent casting and melt extrusion techniques. This cost-effective alternative to traditional, more expensive methods enables the production of hydrophobic PLA filaments, meeting the rising demand for self-cleaning surfaces, particularly in response to the COVID-19 pandemic [121]. Another promising research direction involves PLA-based composites with embedded antibacterial properties through the incorporation of zinc oxide (ZnO) nanoparticles. ZnO enhances antibacterial functionality while maintaining strong mechanical properties, making these composites particularly suitable for biomedical applications such as prosthetics, where infection control is critical. Current studies focus on factors influencing the antibacterial efficacy of PLA-ZnO nanocomposites, including manufacturing parameters and material characteristics. However, challenges remain in improving their processability, thermomechanical stability, and biocompatibility. Addressing these issues is essential for broader adoption in biomedical and other applications requiring effective infection prevention [122].

### 4.4. Ceramics

Ceramics are among the earliest materials used in various applications, with evidence of their use dating back to 22,000 BC [13]. Traditionally, ceramics have been essential in the aerospace industry and are now increasingly adapted for 3D printing. These materials are classified into oxide and non-oxide ceramics, ceramic composites, glasses, and carbon-based ceramics. Common examples such as alumina, zirconia, and silicates are widely used in applications requiring high wear and corrosion resistance, excellent electrical insulation, and superior thermal stability. Carbon-based ceramics, including graphite, diamond, graphene, fullerenes, and CNT, offer distinct properties suitable for diverse applications.

Recent advancements have enabled the adaptation of various additive manufacturing techniques, including Fused Filament Fabrication (FFF), DLP, Stereolithography, Inkjet Printing, SLS, and SLM for ceramic printing [26,123]. However, a major challenge remains in achieving the optimal composition and microstructure for specific applications. The key benefits of 3D printing ceramics include precise porosity control, reduced fabrication time, and the ability to closely match the desired composition. Despite these advantages, challenges such as dimensional inaccuracies, poor surface quality, and the need for extensive post-processing persist [124]. Additive manufacturing of ceramics is gaining attention for its potential in biomedical applications, particularly in developing implants and scaffolds. By designing scaffold structures that replicate bone architecture, 3D-printed ceramics can enhance osseointegration and promote faster healing through tailored structural modifications [13].

### 4.5. Smart Materials

Smart materials react to stimuli like temperature or pressure, making them useful for adaptive applications [84]. These include Shape Memory Alloys (SMAs) and Shape Memory Polymers (SMPs). SMPs recover their original shape when heated, while piezoelectric materials generate electricity under mechanical stress [16]. SMAs are particularly attractive for solving complex engineering challenges due to their high actuation stresses and strains, offering an excellent power-to-weight ratio [125].

#### 4.5.1. SMPs

Due to their adaptability, smart materials are widely used in aerospace [56], biomedical [60], and construction [77] applications, improving efficiency by reducing reliance on complex mechanical systems and enhancing energy performance. Many of these materials, especially biodegradable polymers like PLA, contribute to sustainability by minimizing dependence on petrochemical resources [16,56]. However, their production can be costly due to the complexity of their manufacturing processes, often requiring specialized equipment and controlled conditions.

**Figure 7:**
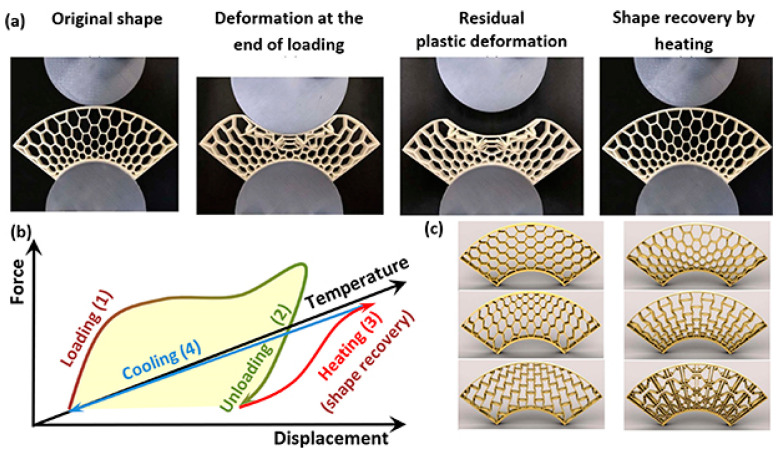
(**a**) Four-dimensional-printed metamaterial before loading and after loading, unloading, and the heating-cooling process; (**b**) schematic diagram of force–displacement–temperature for the SMP metamaterial.; (**c**) different lattice patterns of metamaterials [126].

Lattice-based structures for SMPs and metamaterials developed through 4D printing provide sustainable, eco-friendly solutions. These materials excel in energy absorption, dissipation, shape recovery, and adaptability Figure 7. Their shape memory and reversibility enhance resilience and reusability, reducing energy and material consumption for repairs. The lattice architecture ensures lightweight properties without sacrificing strength. Additionally, these metamaterials function autonomously through thermo-mechanical principles, eliminating the need for complex electronics. A major challenge is meeting key performance requirements, including rapid, controllable activation, high shape-memory efficiency, strength, and durability under cyclic loading [126]. Furthermore, inconsistencies in printed components, such as defects and material variability, can affect thermo-mechanical behavior and shape-memory performance [127]. Addressing these challenges is crucial to ensuring reliability and expanding their practical applications.

Shah et al. highlight the potential of vat photopolymerization (VPP) for producing polymer nanocomposites in smart materials, particularly SMPs with magnetic nanofiller-based composites. These materials offer superior resolution, enhanced magnetic responsiveness, and adaptability to different processing temperatures compared to extrusion or jetting-based AM methods. The researchers propose modifications to VPP systems to stabilize fillers within resins, maximizing the potential of vat polymerization in smart applications [84]. Li et al. identified limitations in self-healing, shape memory, and recyclability in printable polymers. To address this, poly(urethane-urea-amide) (sPUUA) elastomers were developed with dynamic bonds, enhancing self-healing and shape memory properties. These materials exhibit one-way/two-way and multiple-shape memory behaviors, along with superior micro-scratch resistance and thermal self-healing capabilities [127].

SMPs are highly suitable for 4D printing due to their advantages over SMAs: they are easier to manipulate, and their properties can be tailored using simpler methods. Shape recovery can be triggered by various stimuli, including heat, humidity, pH, light, or even a combination of multiple. These characteristics make SMPs the most appropriate material for 4D printing, enabling their use in adaptive and responsive structures [128].

#### 4.5.2. SMAs

**Figure 8:**
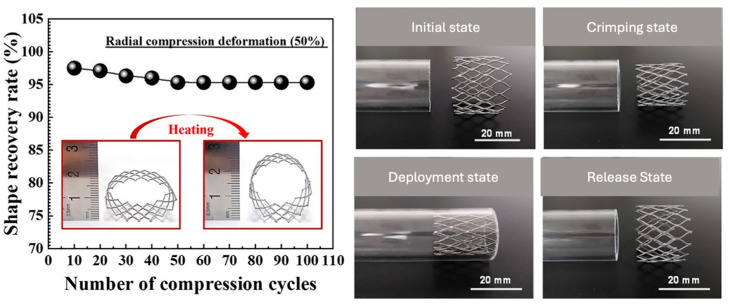
Potential medical application of architected SMA, which could be beneficial by fabricating with AM techniques [130].

SMAs represent a key intersection of materials science and advanced manufacturing. Research by Zafar et al. focuses on refining additive manufacturing techniques, such as selective laser melting, to address challenges in material composition, phase transformation, and mechanical properties [129,130]. Among metallic smart materials, SMAs are capable of undergoing solid-to-solid phase transformations, enabling the shape memory effect and pseudoelasticity [126], enabling 4D printing applications, such as deployable satellite structures and morphing aircraft wings that adjust for optimal aerodynamic performance [125]. A prominent example is Nitinol, a nickel–titanium alloy known for its super elasticity, high biocompatibility, and shape memory effect. These transformations occur between martensite and austenite phases [131] due to thermal or mechanical stimuli, which must be preserved during the printing process through precise control of processing parameters [126,132,133]. Lattice or porous SMA structures offer advantages in specific strength, stiffness, and biocompatibility, making them ideal for applications like stents, where flexibility and recovery are critical. Figure 8 illustrates how LPBF, the most commonly used AM method for SMAs [134], provides high dimensional accuracy, density, and customization. This process supports intricate stent designs incorporating 4D printing techniques, leveraging the shape memory effect to expand or contract in response to external stimuli, such as body temperature [130].

To enhance performance, SMAs can be alloyed with elements such as Cu, Fe, Mn, Al, V, Zr, Ta, Ga, Hf, and Si, as shown in Table 3. These modify mechanical properties, transformation temperatures, and durability.

### 4.6. Biodegradable Materials

The growing demand for sustainable manufacturing solutions has driven research into biodegradable and recycled materials for AM. These materials offer an eco-friendly alternative by reducing environmental impact while maintaining functional performance. Biodegradable polymers decompose under natural conditions, minimizing plastic waste. Meanwhile, recycled materials, including repurposed thermoplastics and metal powders, contribute to circular economy principles by extending material lifecycles and reducing resource consumption. The integration of these materials into AM enables the production of high-performance components with lower carbon emissions, contributing to global sustainability goals. However, challenges such as mechanical property retention, processing optimization, and material availability remain key areas of ongoing research.

Thermomechanical properties of recycled PET, PLA, and ABS, along with agricultural residues, highlight their potential in AM [142]. While these materials support circular economy principles, challenges in mechanical performance, processing stability, and printability persist. Optimizing material formulations, refining recycling methods, and advancing AM processes are crucial for improving their viability. Future research should focus on composite reinforcement and novel biodegradable materials for enhanced AM applications. Other studies analyze the mechanical reliability of biodegradable materials in AM using FDM [143]. Through experimental and Finite Element Analysis (FEA), it examines how defects impact structural integrity. Results show that zero-degree raster orientation enhances strength, while defects increase stress concentration and weaken performance.

New methods are being explored for low-cost VP 3D printing to fabricate biodegradable elastomeric structures using poly octamethylene maleate anhydride citrate (POMaC) [ECO 3]. The VP-POMaC ink, optimized with cross-linkers and porogens, enables high-resolution (80 μm) printing on affordable LCD 3D printers. Mechanical properties can be adjusted through porogen concentration, improving elasticity for biomedical applications. The printed constructs demonstrate 80% cell viability. This approach allows for complex gyroid geometries, contributing to the advancement of biodegradable elastomeric biomaterials [144]. The feasibility of recycled PLA in large-format additive manufacturing (LFAM) using Fused Granular Fabrication (FGF) has been studied [145]. Thermal, rheological, and mechanical analyses reveal that PLA maintains viable properties for up to five recycling cycles despite a gradual decline in molecular weight and viscosity. Compared to filament-based methods, FGF minimizes thermomechanical degradation by reducing reprocessing steps. Demonstrations with 3D-printed components confirm its potential for circular economy applications, reducing plastic waste while preserving material performance.

Conventional recycling techniques, such as melting-based methods, are compared with emerging direct conversion approaches [146], which offer improved material recovery rates and energy efficiency due significance of material waste management, particularly in aerospace, nuclear, and marine industries, where metals such as aluminum, stainless steel, titanium alloys, and superalloys are extensively used. A sustainable approach by integrating recycled aluminum feedstock has been analyzed [147]. Solid-state AM methods, like AFSD, offer advantages over fusion-based techniques by preserving mechanical properties and reducing energy consumption. Recycling pathways, including upcycling and closed-loop recycling, minimize carbon emissions from 14.4 kg CO₂/kg to 0.6 kg CO₂/kg. Despite benefits, challenges in contamination control and property retention require further research.

In studies utilizing carbon fiber acrylonitrile butadiene styrene (CF-ABS) machining waste as a sustainable feedstock for large-format additive manufacturing (LFAM), researchers evaluate the feasibility of recycling CF-ABS machining scraps by repurposing them into pelletized material, addressing concerns related to fiber attrition and degradation [148]. Findings reveal that fiber length is significantly reduced during the recycling process. The results showed an 11% decrease in tensile strength and a 31% reduction in elastic modulus in the print direction—the recycled material unexpectedly shows a 21% increase in tensile strength in the layer-wise direction. The reduction in fiber length lowers viscosity and enhances interlayer adhesion, improving structural integrity despite the mechanical limitations.

Incorporating biodegradable and recycled materials into additive manufacturing promotes sustainability by reducing waste and conserving resources. While advances in polymer recycling and biodegradable elastomers enhance material viability, challenges in printability and mechanical performance persist. In metal AM, recycled aluminum and direct conversion methods lower carbon emissions, but contamination control remains an issue. CF-ABS recycling for LFAM demonstrates improved interlayer adhesion despite fiber degradation. Future research should refine processing techniques and composite reinforcements to maximize performance while maintaining eco-friendly benefits. These innovations will be key to advancing sustainable AM solutions.

## 5. Printing Patterns for Additive Manufacturing

One of the main benefits of 3D printing is its capability to produce models with intricate geometries, made possible by customizable internal structures called infill patterns. These patterns, such as Lines, Triangles, Cubic, Tetrahedral, Concentric, and Zigzag, are designed to optimize printing by reducing material usage and minimizing print time while maintaining structural integrity and a better surface texture [149,150]. FDM process parameters are classified into controllable and uncontrollable categories. The primary factors include built orientation, layer thickness, nozzle diameter, infill pattern, and infill density [150].

**Figure 9:**
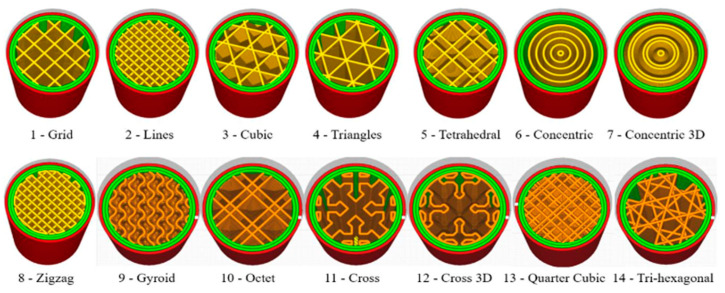
Fourteen infill patterns; open-source software [151].

Most of the 3D infill patterns are labeled as isotropic, meaning that they are equally strong in all directions. Open-source slicer software normally presents 14 infill patterns commonly used, Figure 9, in the research of Pernet et al. tested at varying densities—20%, 40%, 60%, 80%, and 100%—to determine their impact of these 14 infill patterns in terms of their performance under compression and their strength-to-weight ratio [151].

A broad spectrum of materials, including low-temperature metal alloys and composites, are employed in AM techniques; however, thermoplastics and polymer-based composites remain the primary materials utilized in the FDM process. The mechanical properties and quality of an FDM-printed component are greatly affected by process parameters, such as layer thickness, printing speed, nozzle diameter, nozzle temperature, infill density, infill pattern, and build orientation [152]. These parameters must be carefully optimized to achieve a satisfactory product with robust mechanical properties; inadequate temperature settings can result in common printing defects such as warpage and shrinkage, which compromise the structural integrity and dimensional accuracy of the printed object [149].

Researchers highlight the critical role of infill patterns in balancing material efficiency, mechanical strength, and printing speed in 3D printing [10,153]. Common infill patterns, such as lines, offer simplicity and moderate strength, while triangles and cubic patterns enhance stability and load distribution. Tetrahedral infills excel in mechanical performance through three-dimensional interconnectivity, and concentric patterns uniquely manage stress by aligning with model contours. Zigzag patterns, characterized by their wavy structure, prioritize speed and simplicity but may sacrifice strength due to internal gaps that reduce stress-bearing capacity. The strategic selection of infill patterns enables customized trade-offs between efficiency and structural resilience based on application needs. Building on this, Pernet et al. demonstrated that 2D infill patterns like grids, crosses, and triangles outperform 3D patterns in peak load and strength-to-weight ratio. This is attributed to their alignment with the principal stress field and cylindrical axes. Higher infill densities, particularly 80% and 100%, offer superior compressive load support while maintaining excellent strength-to-weight ratios, making them ideal for applications demanding high strength. Conversely, optimized 2D infill patterns are advantageous for lightweight designs requiring material efficiency and structural integrity. These fourteen infill patterns are supported by advanced 3D slicing software and are designed to minimize material usage while maintaining the functionality of the product. Here is a breakdown of these patterns about their benefits and drawbacks that are explained along with their key features described in Table 4.

The most efficient infill patterns significantly impact performance characteristics like speed efficiency, material efficiency, strength-to-weight ratio, structural strength, and versatility. Patterns like Lines and Zigzags are advantageous due to rapid printing and low material consumption, making them suitable for prototyping; however, their minimal Z-axis strength limits their application in load-bearing structures [151]. For applications requiring a high strength-to-weight ratio, patterns like Gyroid and Tetrahedral provide exceptional strength through continuous 3D geometries while optimizing material usage hallmark of these patterns, offering cost and weight savings but often at the expense of structural durability, also demanding longer print times and higher computational resources [150,154]. When maximizing overall structural strength is critical, Octet and Cubic patterns emerge as optimal; these are capable of bearing multidirectional stresses, but their drawback lies in increased material use [155,156]. Cubic and Quarter Cubic infill patterns offer reliable performance across different stress conditions, achieving versatility and balance [157,158]. Among all, Cross 3D and Gyroid infills stand out as the most efficient due to their unmatched strength-to-weight ratio, uniform stress distribution, and adaptability to various designs, and they consist somewhat of slower printing. Their combination of benefits makes them a preferred choice for functional and load-bearing components [151,155]. Cross, Grid, and Triangles are the most efficient due to their high alignment with principal stress directions and lower material usage compared to 3D patterns [151]. Together, these patterns exemplify how tailoring infill pattern designs enhances functionality, efficiency, and customization, but the choice of pattern depends on the intended application.

Future investigations could explore the impact of infill geometry on enhancing the cost-effectiveness and sustainability of 3D printing. Additionally, examining different stress methods, such as tensile and bending tests, and exploring new infill designs could uncover broader applications and refine the utility of these patterns in diverse engineering contexts.

## 6. Technological Trends

The evolution of AM has shifted focus from conventional manufacturing limitations to the creation of intricate extrusion-based additive manufacturing of complex geometries, custom-tailored components, and multifunctional materials, leveraging innovations such as bioinspired structures, machine learning integration, and functionally graded materials. Advancements in biodegradable polymers, mobile AM systems, and artificial intelligence-driven design processes are propelling the technology toward greater sustainability and efficiency. This convergence of innovative materials, scalable systems, and intelligent tools not only broadens the application spectrum of AM but also highlights its pivotal role in fostering environmentally responsible and economically viable manufacturing practices.

### 6.1. Three-Dimensional Micro-Additive Manufacturing

Recent advancements emphasize producing highly complex microstructures for applications in the medical, automotive, optics, and biotechnology industries. The field has shifted toward scalable and hybrid AM systems to overcome the limitations of traditional lithographic and micromachining methods. Innovations such as micro-stereolithography and electrochemical fabrication processes are redefining the production of true 3D micro components, enabling new opportunities in micro-optical systems and integrated microsensors [123,124].

### 6.2. Mobile Additive Manufacturing Systems

These are powered by robotics that are revolutionizing construction by enabling scalable, adaptable, and sustainable on-site fabrication. Unlike traditional fixed gantry systems, MAM offers unbounded workspaces, flexibility for new builds and renovations, and in situ material extrusion, reducing waste and transportation needs. These systems enhance productivity, support sustainable practices, and allow for complex architectural designs and material optimization. By addressing construction challenges and enabling innovative workflows, MAM is driving efficiency, customization, and environmental responsibility in modern construction [124].

### 6.3. Functionally Graded Materials

Techniques such as powder bed fusion and direct energy deposition are optimized for fabricating functionally graded materials (FGMs), which are essential for applications requiring enhanced thermal resistance and mechanical performance. Drawing inspiration from the natural gradients found in biological systems, AM provides precise control over material composition, broadening its utility to fields such as biomedical engineering and sensor development [90]. In the aerospace sector, it is utilized to produce high-strength and lightweight parts with intricate geometries [124] that experience different environmental conditions across their volume.

### 6.4. Artificial Intelligence (AI) and Computer-Aided Design (CAD)

Artificial intelligence (AI) and Computer-Aided Design (CAD) are key drivers of the future of manufacturing. AI enhances design efficiency through intelligent simulations and error reduction, while CAD integrates seamlessly with AM to produce complex geometries and enable material-efficient designs. However, the adoption of AM at an industrial scale is hindered by high costs, material limitations, and process instabilities [159,160]. Emerging trends include identifying high-value applications where AM adds competitive advantage, such as lightweight components or rapid prototyping. These applications push companies to redefine supply chain dynamics, integrating digital workflows and real-time feedback mechanisms. Additionally, AM adoption is transitioning from a single-company perspective to a collaborative, multi-entity ecosystem, emphasizing sustainability, cost efficiency, and technological scalability [161].

#### 6.4.1. Material Innovations for AM

Material development plays a crucial role in overcoming AM challenges. Machine learning (ML) enhances material selection and development, accelerating the creation of advanced composites and alloys; Generative Adversarial Networks (GANs) and Variational Autoencoders (VAEs) enable the generation of new material compositions that optimize mechanical properties and sustainability [162]. Studies indicate that ML-assisted material design can improve classification accuracy in powder selection and optimize microstructural properties for superior mechanical performance [126]. ML is used to analyze scanning electron microscope (SEM) images of AM powders, ensuring consistency in material properties [159].

**Figure 10:**
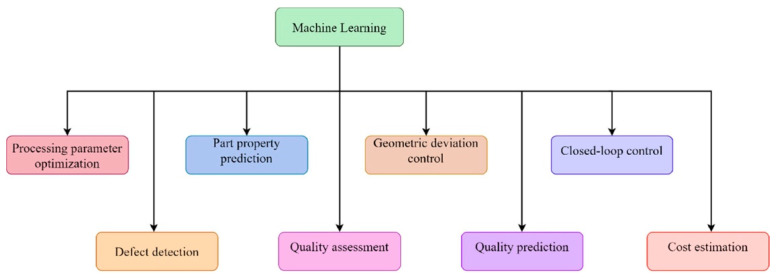
ML innovations in AM [162].

AI can optimize material formulations by predicting the properties of novel composite materials, such as bioinspired structures or FGMs, that enhance mechanical strength while reducing weight [163]. Bayesian Optimization (BO) and Gaussian Processes (GP) help in predicting material behavior under different process parameters, reducing experimental trial-and-error [162]. The integration of bioinspired materials such as nacre-like composites and self-healing polymers enhances AM part durability and energy absorption [164]. ML innovations contribute to a more efficient and sustainable AM ecosystem, Figure 10, improving material utilization rates and reducing post-processing needs and production waste, optimizing parameters like laser power and layer depth to enable desired component properties while minimizing defects via computer vision to improve products and save resources. ML predictive models forecast part performance and shape accuracy, addressing potential issues proactively. Ultimately, combining ML and AI is crucial for advancing AM, ensuring quality, and unlocking cost-cutting opportunities.

#### 6.4.2. Process Control Enhancements

Optimizing AM process parameters is fundamental to ensuring high-quality, defect-free prints while maintaining efficiency. The control of factors such as layer thickness, printing speed, support structure design, and thermal parameters significantly impacts the structural integrity and surface finish of printed components. The AI integration in AM has led to advanced process control methodologies, improving print quality, energy efficiency, and defect detection [162].

Real-time process parameter optimization through closed-loop control systems dynamically adjusts variables such as layer thickness, printing speed, and thermal conditions based on sensor feedback, reducing defects and residual stresses. Bayesian optimization to determine optimal build orientation and layer thickness, reducing support material usage and enhancing print accuracy, minimizing defects, residual stresses, and warping, especially in SLM and EBM processes [164].

ML algorithms, including convolutional neural networks (CNNs) and reinforcement learning models, enhance defect detection and predictive maintenance, enabling early identification of process anomalies. Furthermore, AI-driven CAD optimization integrates finite element simulations to anticipate structural deformations before printing, improving accuracy. The adoption of digital twins and generative AI models optimizes toolpaths and scan strategies, minimizing internal defects and process variability. These AI-based advancements significantly enhance process stability, ensuring consistent, high-quality AM outputs while reducing operational costs and material waste [159,162,164].

#### 6.4.3. Smart Optimization Through AI and CAD Integration

AI-driven CAD optimization enhances the manufacturability and performance of additive manufacturing (AM) parts by integrating generative design, topology optimization, and predictive modeling [159,162].

AI-assisted generative design suggests optimized geometries that minimize weight while maintaining structural integrity, improving material efficiency [162]. Deep learning models, including convolutional neural networks (CNNs) and generative adversarial networks (GANs), facilitate multi-scale topology optimization, leading to more resilient and lightweight components [159,164]. The optimization of multi-material topology in AM improves mechanical performance and material efficiency, while real-time monitoring dynamically adjusts process parameters to minimize waste [163].

AI-powered digital twin technology [162] predicts thermal and mechanical deformations [164], ensuring high-fidelity designs and reducing print errors [159]. To address high costs and inefficiencies in AM, AI-driven optimization techniques refine print parameters and reduce trial-and-error iterations. ML algorithms, such as polynomial regression and differential evolution, optimize layer height, infill density, and support structures for cost-effective production [24]. AI-assisted CAD modeling automates G-code generation, enhancing print path efficiency and minimizing material usage [159,160]. Additionally, neural network models optimize deposition strategies, achieving up to 7.5% improved durability, 11.5% enhanced component thickness, and 4.5% lower manufacturing costs. AI-controlled 4D printing further enables precise manipulation of smart materials, facilitating shape-morphing structures with fewer iterative design cycles. AI-controlled 4D printing further enables precise manipulation of smart materials, facilitating shape-morphing structures with fewer iterative design cycles. Predictive scheduling algorithms enhance machine utilization, reducing downtime and improving overall production efficiency [162]. These AI-driven advancements significantly lower production costs, enhance part quality, and improve the scalability of AM processes.

### 6.5. Laser Additive Manufacturing (LAM)

Advancing materials innovation is essential to fully realize the potential of laser additive manufacturing (LAM), as many current materials, adapted from traditional manufacturing, fall short in LAM’s specialized processes. Research is now focused on designing materials specifically for LAM through interdisciplinary efforts in metallurgy, chemical engineering, and computational modeling. Key advancements include custom alloys tailored to melt pool dynamics for precision and defect reduction, improved powder flowability for consistent layer deposition, and functionally graded materials (FGMs) for site-specific properties inspired by natural gradients. Future efforts aim to prioritize sustainability with recyclable materials and energy-efficient methods while broadening LAM applications to high-stress environments and critical infrastructure [15].

### 6.6. Extrusion-Based Additive Manufacturing (EbAM)

**Figure 11:**
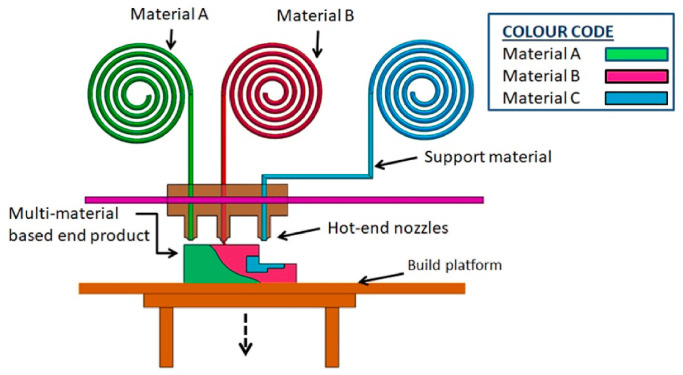
EbAM process of multimaterial-based product with multi-nozzle system [165].

Extrusion-based Additive Manufacturing (EbAM) has emerged as a transformative trend in the field of advanced manufacturing, particularly for the production of multi-material polymeric laminated composite structures; see Figure 11. This technique, which employs layer-by-layer deposition of materials in filament or pellet form, has revolutionized the fabrication of intricate geometries and functional materials with enhanced mechanical properties. Recent advancements underscore its potential in fabricating multi-material laminated composite structures (LCSs) for diverse industrial applications, ranging from aerospace to biomedical engineering. Furthermore, bio-inspired design approaches within EbAM, integrating materials with contrasting properties such as stiffness and flexibility, have broadened their applicability to domains like biomimetics and structural optimization. These innovations highlight EbAM’s capacity to combine materials with distinct mechanical characteristics, enabling the creation of tailored solutions for specific functional requirements [165].

In summary, advancements in AM are revolutionizing industries with precision, efficiency, and sustainability. From intricate 3D microstructures to scalable Mobile Additive Manufacturing (MAM) systems, these technologies address production and construction challenges. Innovations like powder bed fusion, functionally graded materials, and AI-enhanced CAD are transforming design and manufacturing processes. Emphasis on sustainability and materials tailored for laser additive manufacturing (LAM) ensures environmental responsibility and scalability. These breakthroughs redefine innovation and collaboration across diverse industries.

## 7. Technological Inspirations

Nature serves as an inexhaustible source of inspiration, offering ideas primarily categorized as visual (shapes and structures) and functional (single or multifunctionality). Terms such as bionic, bio-inspired, and biomimicry describe approaches that draw on nature’s designs or functions [166].

Biological materials are complex composites with extraordinary mechanical properties despite their seemingly weak components. These structures, shaped by millions of years of evolution, have inspired materials science to design novel materials with features like hierarchical organization, multifunctionality, self-healing, and self-organization, which inspire sustainable innovations across disciplines, fostering interdisciplinary collaboration and technology transfer [13,14]. Bioinspired structures focus on understanding biological systems to address engineering challenges. This involves correlating nature’s principles to practical needs and fabricating hierarchically structured materials that exhibit improved properties [90]. Despite facing current challenges, they are at the forefront of innovation. Bioinspired solutions surpass the limits of conventional engineering materials and significantly contribute to the advancement of the industry.

### 7.1. Biological Structures

Biological structures are naturally optimized to perform specific functions under diverse environmental conditions. These materials often outperform synthetic ones due to their unique design strategies [166].

Wang et al. highlight various biological shapes and structures known for their functional and mechanical properties [14]. Their efficiency lies in their hierarchical organization, spanning multiple length scales from nano- to macro-levels. This architecture allows materials like nacre, spider silk, and bone to achieve a balance of toughness, strength, and low weight, exceeding the capabilities of many homogeneous synthetic materials. Surface textures and patterns are of significant importance in biological functionality, as they influence various interactions at the cellular and molecular levels. For instance, the lotus leaf’s micro- and nano-patterns create superhydrophobic properties, enabling water repellence and self-cleaning. Similarly, the ribbed structure of shark skin reduces drag for improved hydrodynamics, while the grooved design of cactus spines facilitates directional water transport. Adhesion and movement in biological systems rely on specialized structures. Gecko feet use a hierarchical network of setae and spatulae to achieve strong dry adhesion for vertical climbing. Butterfly wings generate vibrant colors through microstructures that interact with light. Biological sensing mechanisms also demonstrate remarkable adaptability: chameleon skin adjusts guanine nanocrystals to change color based on environmental stimuli, spider hairs detect airflow vibrations, and pinecones react to humidity by opening or closing [14,90]. Table 5 summarizes these biological structures and their applications.

**Figure 12:**
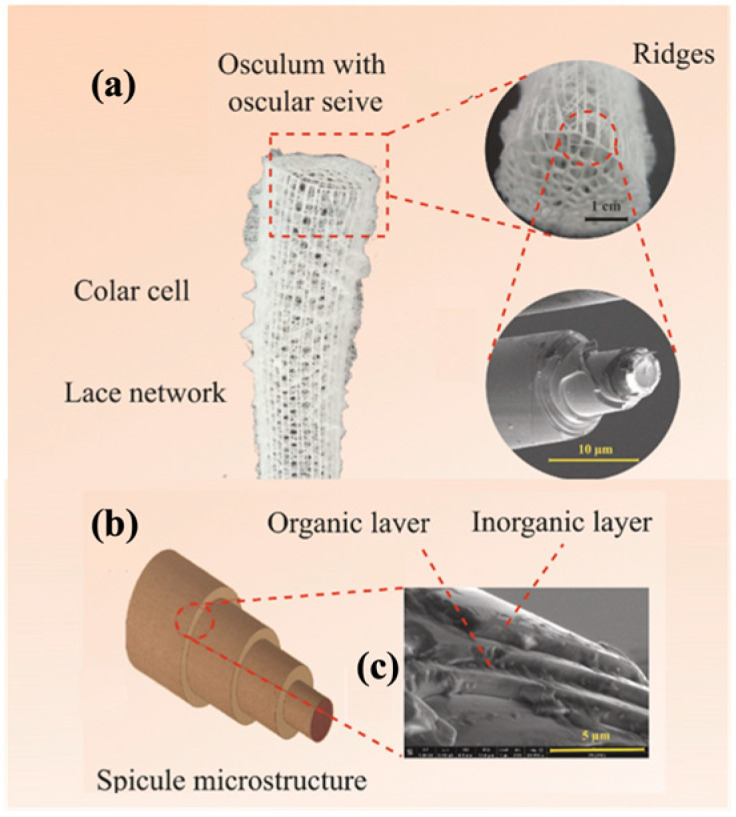
(**a**) Skeleton of Euplectella Aspergillum (EA), (**b**) schematic of spicule structure, (**c**) SEM image of spicule structure showing the alternating concentric cylindrical layers of hydrated silica. Reprinted from the permission with Ref. [167].

One of the most remarkable biological microstructures is found in the marine sponge Euplectella aspergillum (EA), shown in Figure 12. Its exceptional flexural strength comes from an intricate microstructure of concentric cylindrical layers (spicules) interwoven with organic material. This design effectively prevents crack propagation, enhancing mechanical resilience [167].

Overall, the diversity and efficiency of biological structures provide valuable inspiration for developing innovative, sustainable, and high-performance materials [90].

**Table 5:** Biological structures with applications [14,166].

Biological Structure	Nature Serves Function.	Driven Industry	Visual Representation	References
Collagen	Found in bones, tendons, and muscles. Provides tensile strength and structural integrity in tissues.	Healthcare, Biomedicine	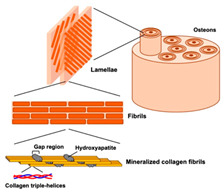	[168,169]
Keratin	Found its protection for hair, nails, horns, and feathers.	Textiles, Construction	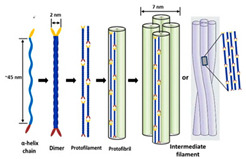	[170]
Chitin	Present in arthropods and insects as protective exoskeletons.	Healthcare, Construction	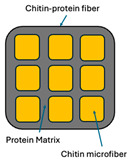	[168,171]
Cellulose	Forms the structural framework (strength) of plant cell walls.	Construction, Other	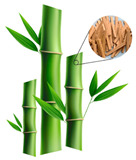	[168,172,173]
Elastin	Found in skin, arteries, and lungs. Provides elasticity and resilience to tissues.	Healthcare	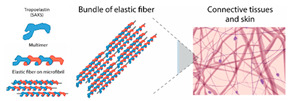	[174,175]
Bone	Composed of hydroxyapatite and collagen. Supports the body structurally and facilitates movement.	Healthcare, Construction	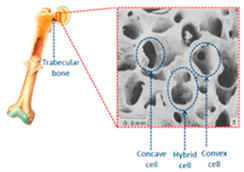	[176,177]
Teeth	Includes enamel and dentin, primarily composed of hydroxyapatite. Facilitates chewing and grinding of food; protects dental nerves.	Healthcare, Dentistry	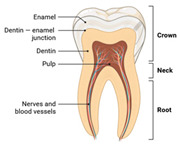	[166,178]
Abalone Shell	Hierarchical structure with aragonite tiles and organic layers. Provides toughness and fracture resistance in marine environments.	Aerospace, Construction	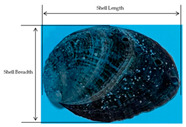	[166,168,179]
Crab Exoskeleton	Composed of chitin-protein fibrils and mineralized components. Combines protection with flexibility in crustaceans.	Construction, Other	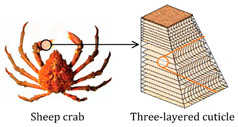	[166,180,181]
Spider Silk	Combines lightweight structure with exceptional tensile strength on strong protein fiber.	Textiles, Aerospace	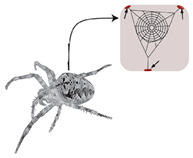	[166,182,183,184]
Mussels’ Byssus	Adhesive and elastic threads for attachment to surfaces in aquatic environments.	Healthcare, Construction	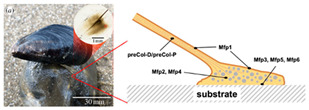	[185,186]
Wood	Cellular material providing support and nutrient transport.	Construction	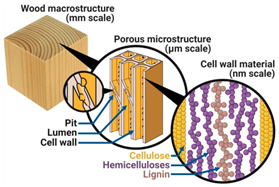	[166,187,188]
Feathers	Lightweight structures with mechanical and thermal properties. Insulates and supports flight in birds.	Aerospace, Textiles	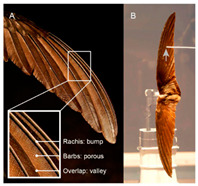	[166,189,190]
Toucan Beak	Composite structure with a foam core and rigid outer shell. Provides lightweight yet strong support for feeding and defense.	Aerospace, Other	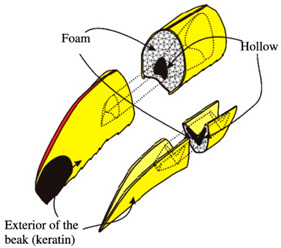	[189,191]
Diatom Shells	Silica-based structures formed via self-assembly in aquatic organisms (*Didymosphenia geminata*)	Healthcare, Construction	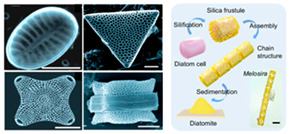	[192,193]
Nacre (Mother-of-Pearl)	Composed of aragonite tiles and organic layers, providing exceptional fracture toughness and durability.	Aerospace, Construction	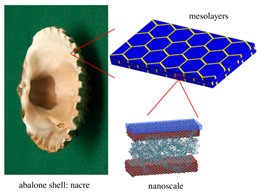	[166,194,195,196]
Cactus Spines	Enable water collection through a hierarchical surface structure.	Healthcare, Construction	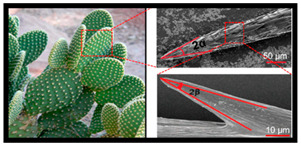	[197]
Crustacean Exoskeletons	Composed of chitin-protein fibrils embedded in a mineralized matrix. Provides structural protection and flexibility.	Construction, Other	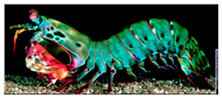	[198,199]
Mammalian Skin	Combines mechanical strength with flexibility.	Healthcare, Textiles	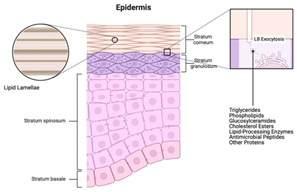	[166,200]
Beehive	Hexagonal cellular structure for efficient space use and strength. Provides structural efficiency and resource optimization.	Aerospace, Construction	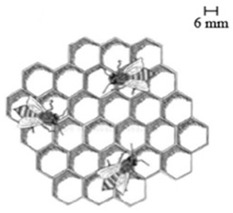	[201]
Cuttlebone	Hierarchical porous and lightweight structure enabling buoyancy, resilience and protection.	Marine, Construction	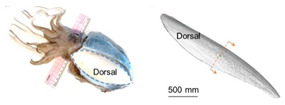	[202,203]
Baleen	Keratin-based structure in whales for filtering food.	Healthcare, Other	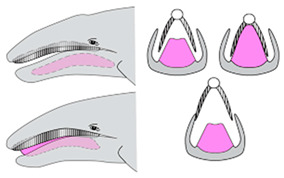	[204]
Iridophores (Chameleon Skin)	Structural coloration through nanocrystals. Provides dynamic color changes for camouflage and communication.	Optics, Other	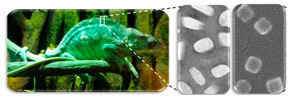	[205]
Pinecone Scales	Bilayer structure, enabling humidity-responsive movement for seed dispersal or protection.	Construction, Other	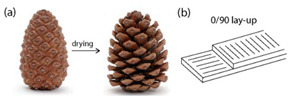	[206,207,208]
Gecko Feet	Hierarchical structure providing strong, reusable adhesion. Enables climbing and attachment on smooth surfaces.	Aerospace, Healthcare	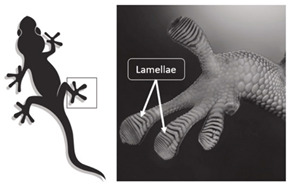	[209,210]
Lotus Leaves	Surface micro textures, offering superhydrophobicity for self-cleaning and efficiency.	Healthcare, Textiles	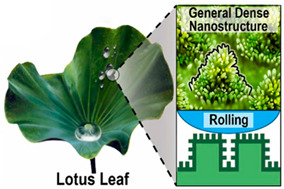	[211]
Butterfly Wings	Microstructures, interacting with light for coloration without pigments.	Optics, Textiles	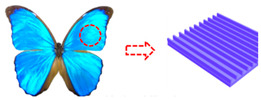	[166,212,213]
Hedgehog Spines	Cellular structure for energy dissipation. Protects against impacts and predators.	Aerospace, Automotive	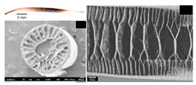	[214,215]

### 7.2. Bioinspired Structures

Inspired by nature, engineers have replicated these biological principles to create advanced bioinspired materials. For example, synthetic superhydrophobic surfaces inspired by lotus leaves are developed for self-cleaning and water-repellent applications. Shark-skin-inspired surfaces with ribbed patterns are designed for drag reduction in marine vehicles. Similarly, cactus-inspired designs utilize gradients in surface structure to facilitate efficient water collection and transport. Bioinspired materials also mimic the hierarchical structures found in natural adhesion mechanisms. Gecko-inspired adhesives replicate the setae and spatulae network to create reusable, strong adhesion pads. Structural coloration seen in butterfly wings has been translated into bioinspired materials for vivid, energy-efficient displays. Bioinspired sensing materials, like the chameleon-inspired mechanically responsive color-shifting elastomers, change colors in response to mechanical stimuli, offering potential applications in displays and sensors. Biological composites such as nacre inspire the development of tough, lightweight materials. Synthetic nacre-like composites leverage the “brick-and-mortar” structure to combine strength and fracture resistance. Similarly, bone’s combination of hard minerals and soft collagen has guided the creation of bioinspired materials for enhanced mechanical properties in medical implants and structural applications [14,90].

### 7.3. Potential Case of Studies

**Figure 13:**
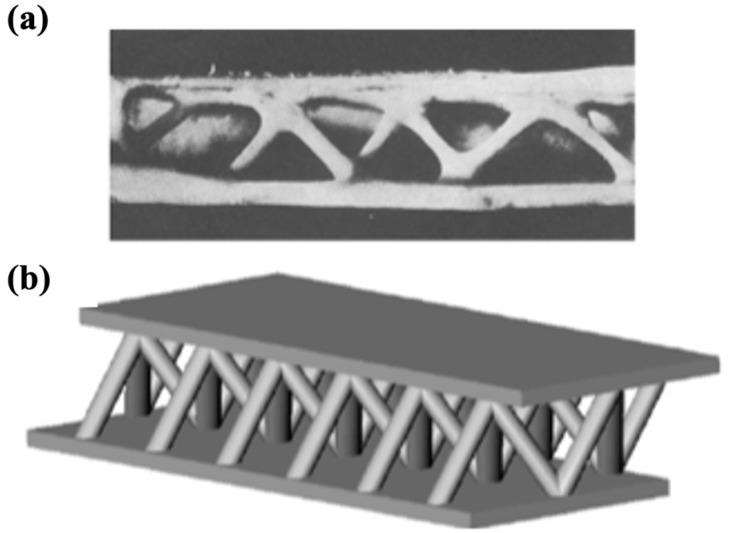
(**a**) Metacarpal bone from a vulture’s wing. (**b**) Stiffened structure supported by V-shaped struts organized in a three-dimensional configuration [166,217].

**Figure 14:**
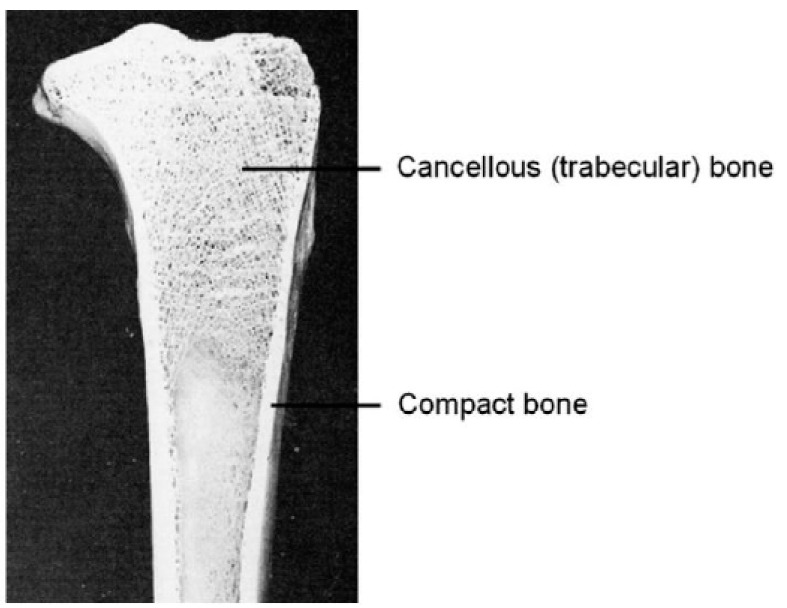
Longitudinal section of tibia [166].

The structural ingenuity of vulture wings [216], as shown in Figure 13, and human bones, as shown in Figure 14, offer a fascinating blueprint for creating lightweight, resilient materials, as studied by Meyers et al. Both systems exemplify how nature achieves an optimal balance between strength and minimal weight, making them valuable inspirations for bioengineering and materials science. The vulture wing’s architecture exemplifies lightweight efficiency. Its metacarpal bone comprises two outer layers connected by a network of angled struts, forming a rigid yet light truss-like structure. This arrangement not only maximizes stiffness but also strategically distributes mass away from the wing’s neutral axis, enhancing its aerodynamic efficiency. This natural design has influenced the development of truss-core materials, which replicate these properties for applications in aerospace and other weight-sensitive fields, and the human bones are marvels of hierarchical engineering. Comprised of collagen and hydroxyapatite, they integrate flexibility with strength through a multi-scale organization. Cortical bone provides dense outer layers for strength, while the porous cancellous bone reduces weight and efficiently distributes loads. These structures exhibit remarkable toughness, with mechanisms such as crack deflection and collagen bridging dissipating stress and preventing catastrophic failure. Additionally, bone adapts dynamically to different stress conditions, further enhancing its durability and resilience [166].

**Figure 15:**
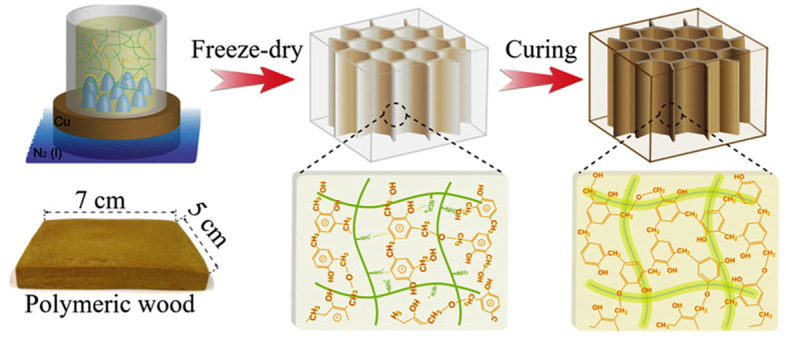
Fabrication route of bioinspired polymeric woods [14].

Wood serves as a remarkable example of a bioinspired material widely utilized in architecture, construction, furniture design, and advanced composite applications. Figure 15, characterized by its highly efficient hierarchical structure, showcases properties such as lightweight, strength, and adaptability due to its multiscale organization, which spans from the nano- to the macro-level. Its architecture combines cellular, fibrous, and porous structures that work synergistically to provide exceptional functionality and allow for exceptional strength-to-weight ratios. Particularly in wood tracheids, it provides anisotropic mechanical properties, making it strong along the grain while maintaining flexibility. This hierarchical structure not only supports efficient load distribution but also ensures resilience and adaptability to environmental stresses. By mimicking wood’s lightweight design and multiscale organization, engineers develop bioinspired materials for construction, aerospace, and sustainable composites [14,166].

Wang et al. highlight that hierarchical structures spanning from the nano- to macro-levels enable a unique combination of lightweight and toughness, as seen in materials like nacre and bone, which achieve high strength and fracture resistance through layered or composite designs that efficiently dissipate energy and withstand mechanical stress. Furthermore, the synergy of hard and soft phases in biological composites provides toughness while maintaining flexibility and a lightweight nature [14].

Lightweight structures with superior mechanical properties are critically required for advanced industrial applications, particularly in areas such as crash mitigation in the automotive and aerospace sectors, due to the outstanding impact energy absorption capability [218]. Marín et al.’s research identified, designed, and optimized a thin-walled structure for energy absorption applications.

**Figure 16:**
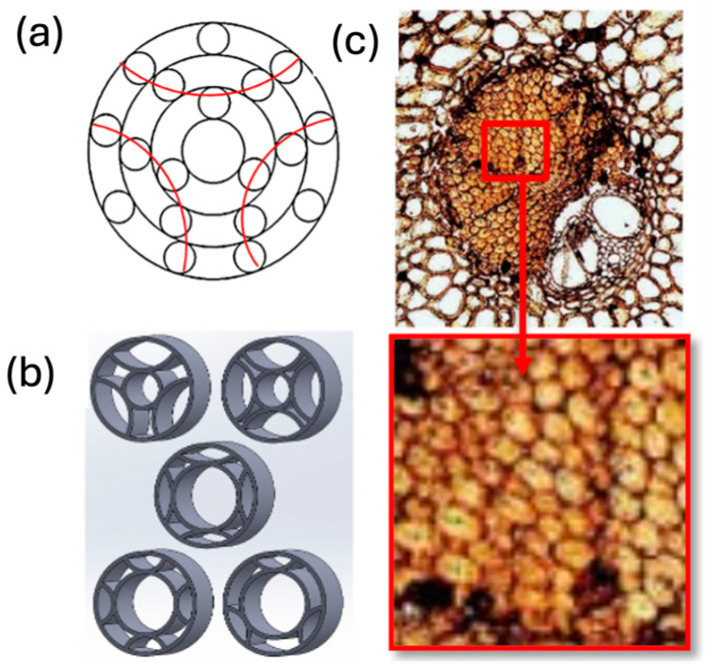
(**a**) Reference geometry of the coconut palm, (**b**) bio-inspired structure arrangements of cylindrical thin-walled structures for energy absorption, (**c**) multicellular anatomy structure of vascular bundle system of the coconut palm, enlarged view for looking multicellular microstructure (red box) [219].

The structure is inspired by the anatomy of the coconut palm (Cocos nucifera); see Figure 16. It serves as a remarkable source of inspiration for bioinspired structural designs, leveraging its unique anatomical features to address engineering challenges. The stem’s circular cross-section, characterized by a gradient distribution of dispersed vascular bundles embedded in supportive ground tissue, functions as a natural reinforcement system, enhancing mechanical stability and load-bearing capacity. This hierarchical arrangement employs gradients in density and thickness to optimize mechanical performance, a strategy that effectively manages structural forces and resists deformation. By mimicking these natural design principles, such as the thin-walled cylindrical structure and the energy-absorbing capabilities of vascular bundles, bioinspired innovations achieve lightweight yet robust configurations with exceptional strength-to-weight ratios. These designs not only enhance energy dissipation in applications like impact resistance and vibration mitigation but also promote sustainability by drawing inspiration from renewable, biodegradable resources. The adaptability and efficiency of these biomimetic structures highlight their potential for diverse applications, including protective gear, building materials, and transportation systems, where resilience and energy absorption are paramount to innovate in structural engineering applications.

**Figure 17:**
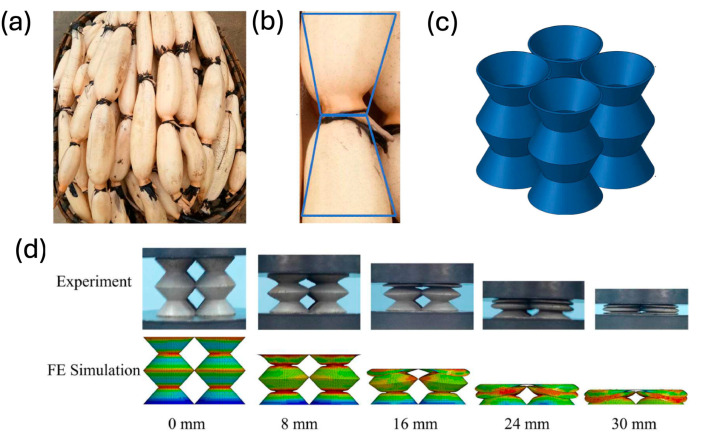
(**a**) Lotus roots; (**b**) enlarged view of lotus roots with their contours highlighted by blue lines; (**c**) schematic diagram of a unit cell of BLRLS and 2 × 2 × 2-unit cells; (**d**) comparison of the experimentally observed and FE simulated deformation history of BLRLS [220].

Xu et al. highlight the biomimetic lotus root lattice structure (BLRLS) evaluation for its mechanical properties, energy absorption, and crashworthiness in real-world applications [220]; Figure 17. The BLRLS demonstrated a specific energy absorption (SEA) of 984.84 kJ/kg, which is significantly higher than alternative multi-cell structures; it was manufactured using AlSi10Mg via selective laser melting (SLM). The mean crushing force (MCF) reached 719.44 kN, approximately 20 times that of a square tube structure. Experimental testing and FEA were performed. The deformation process was gradual, with an orderly and controllable crushing sequence from top to bottom layers, contributing to its high energy absorption capability using an INSTRON 1342 electro-hydraulic servo universal testing machine at 2 mm/s loading speed. The simulation error between theoretical and experimental crash performance indicators was 2.68% for IPCF, 0.52% for EA, and 3.54% for MCF, confirming the reliability of FEA for crashworthiness analysis. BLRLS exhibits exceptional energy absorption, lightweight properties, and high mechanical strength, making it highly suitable for engineering applications requiring impact resistance, such as automotive crash structures, aerospace safety components, and protective barriers [220].

**Figure 18:**
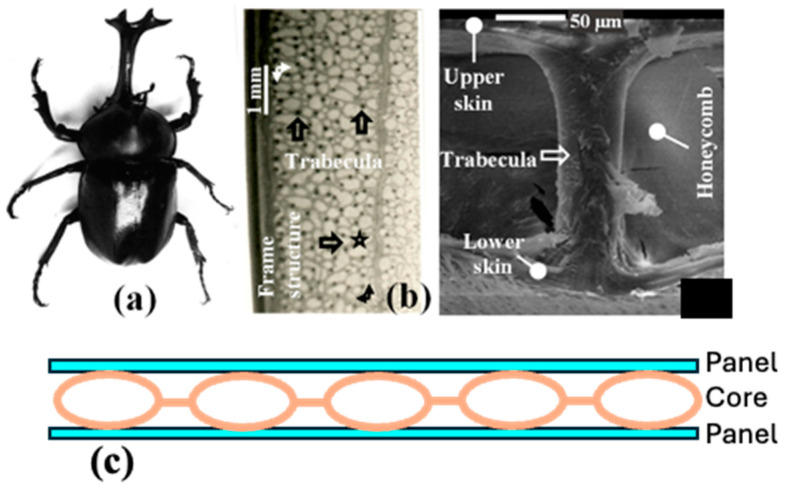
Beetle-inspired structure, (**a**) an adult beetle of Allomyrina dichotoma, (**b**) beetle’s trabecular structure, (**c**) beetle-inspired sandwich structure with an array of cavities [221].

The beetle-inspired structure in Figure 18 exhibits higher energy absorption due to increased internal connections. The hierarchical arrangement leads to improved crashworthiness compared to conventional hexagonal honeycombs. A bi-tubular thin-walled structure based on beetle elytra enhances energy dissipation, contributing to superior impact resistance and deformation control. Forewings of beetles can withstand punch loads up to 23 N, and the hierarchical trabecular structures contribute to 30 times higher inter-laminar strength compared to pure chitin fiber laminas. Crashworthy lattice structures inspired by beetle forewings show superior energy absorption under axial loads, and numerical modeling confirms their advantage over traditional crash box designs. Beetle-inspired structures exhibit multi-scale hierarchical architectures with irregular cellular patterns and trabecular (pillar-like) reinforcements but pose difficulties in replicating them precisely using AM techniques. The anisotropic mechanical properties of beetle-inspired structures require careful process control; layer-by-layer deposition can introduce inconsistencies, leading to weaker interlaminar bonding compared to natural structures. Despite these challenges, this structure is a promising candidate for crashworthy and protective applications [168].

**Figure 19:**
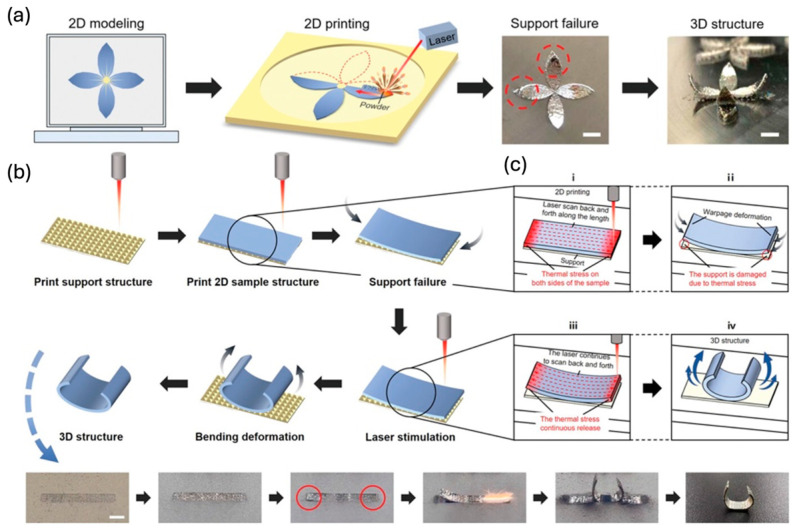
(**a**) Schematic representation of LPBF-based metallic 4D printing through pre-set thermal laser, (**b**) overall process of the triggered shape morphing samples, (**c**) impact of laser scanning strategies [222].

Printing with laser stimulation enables shape-morphing capabilities; unlike traditional methods that rely on SMAs, this approach programs thermal stress into the structure during the LPBF process, allowing deformation upon laser-induced stress release, Figure 19. Key quantitative performance parameters are evaluated as laser power of 250 W providing energy for material fusion, scanning speed of 1600 mm/s for efficient processing without excessive heat accumulation; layer thickness of 40 μm and hatch distance of 50 μm contributing to fine structural resolution, and a thickness of 0.4 mm. Printing with laser stimulation successfully replicates biological structures, such as flowers, mimosa leaves, frog tongues, dragonfly wings, and butterfly wings, mimicking natural movement. This technique demonstrates on-demand shape correction and can be applied to self-morphing components in satellites, reducing dependency on traditional repairs in space missions. Additionally, it can be adapted for customized implants or surgical devices with tunable mechanical properties. While this method significantly advances metallic 4D printing applications, further investigation is required to enhance the service life and mechanical stability of these structures in engineering environments, extend the methodology to non-shape-memory metals, and explore industrial scalability for high-performance applications [222].

Additive Manufacturing (AM) has increasingly drawn inspiration from natural structures to enhance the mechanical properties and functionalities of fabricated parts. Recent studies have explored various bio-inspired designs across the seven AM categories, as shown in Table 6:

## 8. Conclusions

The transformative impact of additive manufacturing is evident in its ability to produce complex, lightweight structures while minimizing material waste and energy consumption through customizable designs and tailored properties. The technological evolution of AM, material innovations, and the integration of bioinspired designs that mimic natural structures enhance performance and demonstrate increasing versatility across diverse industries. This section synthesizes key findings, trends, recommendations, limitations, and potential future applications of AM.

AM has progressed from rudimentary processes, such as photosculpture, to advanced techniques, including PBF and DED. These advancements offer significant benefits, such as reduced material waste, energy efficiency, and the capability to fabricate complex, lightweight structures unattainable through traditional subtractive methods. The broad range of AM-compatible materials, including metals, polymers, composites, and smart materials, has significantly expanded its applications. Innovations in multi-material printing and bioinspired designs have enhanced precision, speed, and cost-efficiency. Binder jetting and hybrid approaches further extend AM’s applicability, enabling the production of intricate, high-performance components across aerospace, biomedical, and industrial sectors. Research into biodegradable and sustainable materials aims to amplify AM’s environmental benefits while addressing limitations in material properties.

Current advancements, including AI integration, digital twin technology, and robotic-assisted AM, are transforming the manufacturing landscape. These innovations enhance efficiency, precision, and sustainability while enabling real-time optimization of designs and processes. Industrialization of AM is shifting from prototyping to large-scale production, incorporating automation, process control, and digital technologies.

Standardized testing and certification protocols must be established to ensure reliability and regulatory compliance across industries. Interdisciplinary collaborations will drive the integration of AM with advanced technologies, such as robotics, to enhance efficiency and adaptability. Additionally, promoting modular and distributed manufacturing systems will facilitate localized, scalable production, expanding the accessibility and impact of AM.

## 9. Challenges and Limitations

Despite its transformative potential, additive manufacturing (AM) still faces several bottlenecks that hinder its widespread adoption. Among the most pressing challenges is high cost, as AM remains expensive due to material expenses, process inefficiencies, and the need for extensive post-processing. The limited availability of AM-compatible materials further restricts its applications, particularly in multi-material printing and functionally graded structures. Inconsistent process outcomes present reliability concerns, as variability in mechanical properties and print quality affects the repeatability of AM processes.

The increasing demand for sustainable AM solutions has driven research into biodegradable and recycled materials. Biodegradable polymers offer a solution to plastic waste, while recycled materials support circular economy principles by extending material lifecycles. However, environmental concerns related to AM, including high energy consumption, emissions, and waste management, require immediate attention.

Scalability remains a critical challenge as AM continues to struggle with transitioning from prototyping to high-volume production. This issue is particularly evident in the fabrication of thin-walled ultra-high vacuum (UHV) components, requiring further research to optimize processing parameters, particularly in metal PBF applications.

To fully harness the potential of AM, material innovation must be prioritized, with the development of cost-effective, high-performance materials. Additionally, interdisciplinary collaborations and the integration of AI, big data analytics, and robotics will be essential to improving efficiency, adaptability, and scalability. Overcoming these barriers is crucial for positioning AM as a sustainable, scalable, and high-performance manufacturing technology.

## 10. Future Perspective

The future applications of AM span multiple industries, underscoring its transformative potential. In healthcare, AM is driving advancements in precision medicine by allowing the creation of customized implants, tailored prosthetics, and bioprinted tissues designed for individual patients. The integration of biocompatible materials and multi-material printing is advancing the production of medical devices, improving functionality and patient compatibility. In the aerospace and automotive sectors, the fabrication of lightweight, high-strength components enhances fuel efficiency and reduces costs, while bioinspired designs and functionally graded materials improve structural integrity under extreme conditions. AM also contributes to sustainability, with biodegradable materials and circular manufacturing practices driving progress toward environmental objectives. Additionally, the implementation of cutting-edge technologies, such as AI and IoT, is crucial for optimizing design processes, facilitating predictive maintenance, and enhancing the efficiency of smart manufacturing systems.

Future advancements in AM must address current limitations by improving material properties, optimizing printing processes, and integrating emerging technologies. The development of bioinspired designs, functionally graded materials (FGMs), and hybrid manufacturing systems presents promising pathways for innovation. AI and CAD remain key drivers in AM, enhancing design efficiency through intelligent simulations and error reduction. AI-driven CAD optimization integrates generative design, topology optimization, and predictive modeling, reducing material waste while improving structural performance. The transition from isolated AM adoption to a collaborative, multi-entity ecosystem further supports sustainability, cost efficiency, and scalability.

In the next 5–10 years, intelligent AM and 4D printing will revolutionize manufacturing across industries, from aerospace to healthcare, by enabling autonomous, self-optimized production with minimal waste. Although challenges remain, the integration of machine learning, smart materials, and digital twins will accelerate AM’s path toward mass industrialization and real-world applications.

## Figures and Tables

**Table 1:** A summary of these seven AM techniques printing materials, applications, benefits, and drawbacks [30,56,97].

Methods	Materials	Applications	Benefits	Drawbacks	Printing Resolution Range (Z-Direction)	Maximum Printing Envelope, (L × W × H)
Binder Jetting	Metals: Stainless steel Polymers: ABS, PA Ceramics: Glass	Foundry industries Biomedical	Faster technique No melting Average mechanical strength Low durability	Lack of adhesion between layers Coarse-resolution Time-consuming postprocessing.	13–16 μm	1800 × 1000 × 700 mm (e.g., ExOne X1 160Pro) [49].
Directed Energy Deposition	Metals: Cobalt, Chrome, Titanium, Nickel	Aerospace Biomedical	Reduces time and cost Extraordinary mechanical properties Grain structure manipulation Accurate printing	Limited material use. Expensive. Post-processing finishing	250 μm	Potentially unlimited [65].
Material Extrusion	Polymers: ABS, Nylon, PC, PLA	Rapid prototyping Toys Advanced composites	Low-cost High-speed printing Easiness Average mechanical properties.	Limited materials range. Less accurate. Restricted build volume. Low printing speed.	50–200 μm	1000 × 1000 × 1000 mm (e.g., BigRep ONE) [61].
Material Jetting	Polypropylene, HDPE, PS, PMMA	Prototyping Biomedical	Wasteless Thinner printing layers High accuracy	Weak mechanical properties. Lack of adhesion between layers.	5–200 μm	490 × 390 × 200 mm (e.g., Stratasys J750) [98].
Power Bed Fusion	SHS: Nylon DMLS, SLS SLM: Stainless Steel, Titanium, Aluminum	Lightweight structures. High performance. engineering parts	High-quality printing. Fine resolution Good mechanical properties	Size limitations. Costly. High porosity.	250 μm	700 × 380 × 580 mm (e.g., EOS P 770 for SLS) [99].
Sheet Lamination	Polymer composites Ceramics Paper Metal-filled tapes Metal rolls	Paper industry Electronics Smart structures Ultrasonic welding	Ability to produce larger structures. Inexpensive process. Environmental friendless.	Complex shapes limitations. Low dimensional accuracy and strength. Frequency bonding of different materials	Variable thickness depends on laminates.	1000 × 800 × 500 mm (e.g., Fabrisonic SonicLayer 7200) [100].
Vat Polymerization	Polymers: UV-curable Photopolymer resin	Medical and dental industries	Better finish High quality	Slow printing High-cost printing setup	10 μm	1500 × 750 × 500 mm (e.g., 3D Systems ProX 950) [101].

**Table 2:** Summary of metals and alloys with their applications and mechanical properties [9].

Materials	Applications	Mechanical Properties	Ultimate Tensile Strength (UTS)	Yield Strength (YS)
Stainless Steel 316	Structural components in construction, automotive exhaust systems, aerospace brackets, food-grade equipment.	High strength, excellent corrosion resistance, suitable for high-stress environments.	450–705 MPa	415–590 MPa
Aluminum Alloys AlSi10Mg	Aerospace fuselage components, automotive engine parts, lightweight frames, heat exchangers.	Low density, good corrosion resistance, moderate strength.	350–450 MPa	200–300 MPa
Nickel Alloys IN718	Turbine blades, jet engine components, combustion chambers, heat shields.	High temperature resistance, excellent fatigue performance.	414–941 MPa	352–580 MPa
Cobalt-Chromium Alloys Co-Cr-Nio HEA	Dental implants, orthopedic joints, cranial plates.	High wear resistance, biocompatibility.	730–840 MPa	500–620 MPa
Titanium Alloys Ti-6Al-4V	Aerospace engine casings, structural airframe parts, biomedical hip and knee implants.	High strength-to-weight ratio, excellent biocompatibility.	900–1100 MPa	800–1000 MPa
Tool Steels H13	Injection molding tools, cutting tools, dies for metal forming.	High hardness, wear resistance.	1300–1600 MPa	1000–1200 MPa
Copper Alloys	Electrical connectors, cooling plates, heat exchangers, rocket engine nozzles.	High thermal and electrical conductivity.	200–300 MPa	100–200 MPa

**Table 3:** Summary of properties and applications of alloying elements in SMAs.

Element	Properties	Impact on SMAs	Applications	References
Copper (Cu)	Lowers transformation temperatures, enhances thermal stability, improves corrosion resistance, and reduces production costs.	Used in Cu-Al-Ni, NiTiCu, and alloys produces exceptional SME with recoverable strains over 50%	Various, especially where lower activation temperatures are needed Aerospace actuators and sensors.	[134,135]
Iron (Fe)	Improves corrosion resistance and enhances stability at high temperatures. Modifies transformation behavior and improves fatigue resistance.	Produces NiTiFe alloys, suitable for high-temp applications. Fe-Mn-Si for smart structures.	Aerospace actuators and other high-temp applications. Smart structures, bridges, and damping systems.	[134,136]
Manganese (Mn)	Enhances shape memory properties and decreases hysteresis. Refines grain structure and stabilizes martensitic transformation	Allows temperature adjustment during transformation, improves biocompatibility	Medical applications, others requiring precise temperature transformations	[134]
Aluminum (Al)	Increases mechanical strength, improves resistance to attrition Reduces density for lightweight applications,	Used in NiTiAl alloys for high mechanical performance applications	Robotics, medical devices implants. Seismic applications. aerospace, automotive, and lightweight	[134,137]
Vanadium (V)	Enhances mechanical properties and high-temperature stability. Strengthens the SMA matrix, improves wear resistance, and refines microstructure.	Used in high-strength NiTi-V. Used in high-temp applications, affects phase transformation temperatures	Fire safety systems, automotive components	[134,138]
Zirconium (Zr)	Decreases hysteresis, enhances corrosion resistance and increases work output	Found in NiTi-Zr. Enhances superelasticity and shape memory characteristics of NiTiZr alloys	Medical and aerospace applications	[134,139]
Tantalum (Ta)	Exceptional corrosion resistance, high strength	Potential for high-temperature SMAs; affects fatigue life	Orthodontic wires, medical implants	[134]
Gallium (Ga)	Low-temperature actuation capabilities. Increases ductility and enhances shape recovery properties.	Used in NiTi-Ga. Suitable for self-healing materials and microscale actuators	Applications requiring low-temp actuation	[134,140]
Hafnium (Hf)	Enhances high-temperature performance and phase stability	Used in high-temp applications, improves surface integrity and abrasion resistance	High-temperature stability required applications	[134]
Silicon (Si)	Improves mechanical properties, enhances resistance to attrition	Found in Fe-Mn-Si-based and used in NiTiSi alloys for applications requiring high mechanical strength	Robotics, aerospace applications	[134,141]

**Table 4:** Overview of 3D printing infill patterns [151].

Infill Pattern	Grid	Lines	Cubic	Triangles	Tetrahedral	Concentric	Concentric 3D
Description	Crisscross pattern with perpendicular lines forming squares.	Parallel lines printed along the X or Y-axis in each layer.	3D grid of cubes oriented with one corner down, creating air pockets.	Interconnected triangles forming a honeycomb-like structure.	Stacked tetrahedrons for supporting vertical pressure and stress.	Traces model perimeters, creating concentric shapes toward the center.	Extends the concentric pattern into three dimensions.
Key Feature	Moderate strength, economical material usage, suitable for general-purpose prints.	Fast to print, uses minimal material, ideal for decorative and calibration prints.	Solid strength, good insulation properties, moderate print time, and material usage.	Excellent structural integrity evenly distributes forces, strong and durable.	High strength, increased material usage, longer print times.	Flexible, allows stretching and twisting, good for flexible parts.	Additional support and strength, suitable for complex geometries.
Benefits	High strength-to-weight ratio; performs well under compressive loading.	Maximizes tensile strength when aligned with load direction.	Good for tensile and flexural applications; consistent stress–strain relationships.	Excellent compressive strength and rigidity.	Isotropic strength; ideal for complex geometries.	Efficient under compressive loads; high strength-to-weight ratio.	Provides isotropic strength, suitable for multi-directional loads.
Drawbacks	Limited for isotropic stress conditions; 2D nature restricts versatility.	Less effective for multi-directional loads.	Lower strength-to-weight ratio than simpler patterns.	Less effective in tensile applications.	High material consumption compared to 2D patterns.	Limited in applications needing isotropic support.	Lower strength-to-weight ratio compared to 2D patterns.
**Infill Pattern**	**Zigzag**	**Gyroid**	**Octet**	**Cross**	**Cross 3D**	**Quarter Cubic**	**Tri-Hexagonal**
Description	Continuous, uninterrupted line forming a zigzag pattern per layer.	Alternating cresting waves in 3D layers.	Similar to tetrahedrals, stacked tetrahedrons provide strength and stress resistance.	Grid of crosses with hollow centers.	3D angled version of Cross pattern for more rigid parts.	Offset pyramidal shapes combining Cubic and Octet elements.	Mix of large hexagons and smaller triangles forming star-like patterns.
Key Feature	Fast to print, low material usage, lower strength, suitable for decorative items.	Excellent strength in all directions, great for flexible but slightly rigid parts.	High strength, good vertical pressure resistance, uses more filament.	Retains structural integrity under stretching, bending, and twisting, suitable for flexible parts.	Slightly more rigid than standard Cross infill.	Strong with good bonding, uses more material.	Balanced strength and material usage, economical for parts needing moderate resistance to force.
Benefits	Moderate strength-to-weight ratio; efficient at lower densities.	Excellent isotropic strength; smooth strain distribution; ideal for complex geometries.	High structural rigidity; ideal for isotropic stress scenarios.	High strength-to-weight ratio for compressive loads among 2D patterns.	Enhanced isotropic properties for complex loads.	Suitable for lightweight and moderately complex geometries.	High strength-to-weight ratio; efficient for compressive loading.
Drawbacks	Inferior compressive strength compared to uniform patterns.	Lower compressive strength than 2D patterns.	High material usage; less efficient for unidirectional loads.	Limited tensile alignment; less effective for multidirectional loading.	Uses more material than simpler patterns.	Lower strength-to-weight ratio compared to simpler patterns.	Limited applicability in tensile or isotropic loading scenarios.

**Table 6:** Current bio-inspired structures in additive manufacturing across seven AM methods.

Additive Manufacturing Method	Bio-Inspired Structure	Purpose	Visual Representation	Reference
Material Extrusion (ME)	Spider web-inspired structures, bamboo-like lattices, and hollow tubes.	Utilizing the hierarchical and heterogeneous design of structures with enhanced toughness, impact resistance, and flexibility.	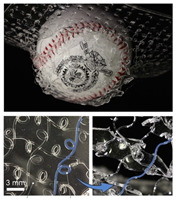 Spider web-inspired structure	[182,183]
Binder Jetting (BJ)	Nacre (mother-of-pearl) and Bouligand structures:	Enhances impact resistance and energy absorption through hierarchical organization. Mimicking the helicoidal arrangements found in mantis shrimp’s dactyl.	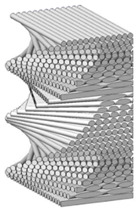 Bouligand structure	[223]
Directed Energy Deposition (DED)	Bone-like structures and porous structures.	Provides a high strength-to-weight ratio and material efficiency.	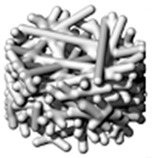 Porous structure	[224]
Material Jetting (MJ)	Squid beak gradient structures, lotus leaf-inspired hydrophobic surfaces	Enables parts with varying mechanical properties, improving functionality.	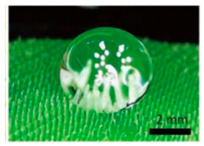 Hydrophobic lotus leaf	[225]
Powder Bed Fusion (PBF)	Bird bone-like lightweight structures. Natural cellular materials (metamaterials)	Optimizes strength-to-weight ratios and incorporates internal lattices for weight reduction. Natural cellular materials to achieve ultralight and ultrastiff properties.	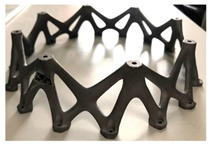 Lightweight structure	[226,227]
Sheet Lamination (SL)	Nacre-inspired brick-and-mortar arrangements	Enhances toughness and impact resistance in laminated composites.	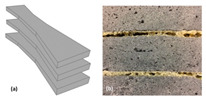 Brick-mortar structure	[228]
Vat Photopolymerization (VP)	Wood-like microstructures, porous networks, shark skin-inspired drag-reducing surfaces	Creates lightweight, strong components with applications in fields like biomedical engineering.	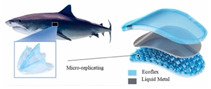 Shark skin-inspired surface	[229]

## Data Availability

No new data were created or analyzed in this study.
